# Association of leptin and leptin receptor gene polymorphisms with systemic lupus erythematosus in a Chinese population

**DOI:** 10.1111/jcmm.13093

**Published:** 2017-02-28

**Authors:** Hong‐Miao Li, Tian‐Ping Zhang, Rui‐Xue Leng, Xiang‐Pei Li, De‐Guang Wang, Xiao‐Mei Li, Dong‐Qing Ye, Hai‐Feng Pan

**Affiliations:** ^1^ Department of Epidemiology and Biostatistics School of Public Health Anhui Medical University Hefei Anhui China; ^2^ The Key Laboratory of Major Autoimmune Diseases, Anhui Province Hefei Anhui China; ^3^ Department of Rheumatology Anhui Provincial Hospital Hefei Anhui China; ^4^ Department of Nephrology The Second Affiliated Hospital of Anhui Medical University Hefei Anhui China

**Keywords:** leptin, single‐nucleotide polymorphisms, systemic lupus erythematosus

## Abstract

To explore the association of *LEP* and leptin receptor (*LEPR*) gene single‐nucleotide polymorphisms (SNPs) with susceptibility to systemic lupus erythematosus (SLE) in a Chinese population. Four *LEP* SNPs (rs11761556, rs12706832, rs2071045 and rs2167270) and nine *LEPR* SNPs (rs10749754, rs1137100, rs1137101, rs13306519, rs8179183, rs1805096, rs3790434, rs3806318 and rs7518632) were genotyped in a cohort of 633 patients with SLE and 559 healthy controls. Genotyping of SNPs was performed with improved multiple ligase detection reaction (iMLDR). No significant differences were detected for the distribution of allele and genotype frequencies of all 13 SNPs between patients with SLE and controls. The genotype effects of recessive, dominant and additive models were also analysed, but no significant evidence for association was detected. However, further analysis in patients with SLE showed that the TT genotype and T allele frequencies of the *LEP* rs2071045 polymorphism were nominally significantly higher in patients with pericarditis (*P* = 0.012, *P* = 0.011, respectively). In *LEPR*, the GA/AA genotype and A allele frequencies of the rs1137100 polymorphism were both nominally associated with photosensitivity in patients with SLE (*P* = 0.043, *P* = 0.018, respectively). Moreover, the genotype and allele distribution of rs3806318 were also nominally associated with photosensitivity in patients with SLE (*P* = 0.013, *P* = 0.008, respectively). No significant differences in serum leptin levels were observed in patients with SLE with different genotypes. In summary, *LEP* and *LEPR* SNPs are not associated with genetic susceptibility to SLE, but may contribute to some specific clinical phenotype of this disease; further studies are necessary to elucidate the exact role of *LEP* and *LEPR* genes in the pathogenesis of SLE.

## Introduction

Systemic lupus erythematosus (SLE) is a systemic autoimmune disorder involving multiple organs and tissues such as skin, kidneys, joints, lung and central nervous system. The aetiology and pathogenesis of SLE generally considers an involvement of environmental factors, which could trigger abnormal autoimmune responses in individuals who carry a predisposing genetic background 1. Therefore, genetic variants within immune‐modulating genes may confer the risk of SLE.

Leptin is an adipokine that plays a key role in the modulation of immune responses and the development and maintenance of inflammation 2. It is a 16‐kD non‐glycosylated polypeptide hormone mainly produced by white adipose tissue (WAT), encoding by the *obese (ob)* gene of murine homolog of human *LEP* gene 3. Investigations have shown that leptin is increased during acute infection and inflammation, indicating that leptin acts as a pro‐inflammatory cytokine. It enhances macrophage phagocytosis activity and stimulates them to produce several pro‐inflammatory cytokines, such as IL‐1, IL‐6 and TNF‐α 4. Leptin exerts its biological actions through the activation of leptin receptor, which belongs to the class 1 cytokine receptor superfamily and are encoded by the *diabetes (db)* gene 5. Numerous studies have shown abnormal increase in serum/plasma leptin levels in patients with SLE 6, 7, 8, 9, but the data regarding association between leptin‐related gene polymorphisms and SLE is still very limited. Afroze *et al*. 10 showed that a *LEPR* gene polymorphism (*LEPR Q223R*) was associated with SLE susceptibility in a Kashmiri population. A recent study assessed *LEP* and *LEPR* gene polymorphisms in four different ancestral groups with SLE, but the results did not support associations between leptin‐related polymorphisms and increased SLE susceptibility 11. In this study, we carried out a case–control study to explore whether *LEP* and *LEPR* gene polymorphisms are associated with SLE susceptibility in a Chinese population.

## Materials and methods

### Study participants

A total of 633 patients with SLE were recruited from the Department of Rheumatology and Immunology at the First Affiliated Hospital of Anhui Medical University, Anhui Provincial Hospital and Anqing Hospital Affiliated to Anhui Medical University. All patients met the 1997 American College of Rheumatology (ACR) revised criteria for the classification of SLE 12. The disease severity was quantified according to the SLE disease activity index 2000 (SLEDAI‐2K) 13. More active SLE was defined as a SLEDAI‐2K score >10, and those patients with SLEDAI‐2K ≤10 were classed as relatively inactive 14, 15. Normal controls were recruited from the general population and healthy blood donors and were geographically and ethnically matched with patients with SLE. All the normal controls did not have a history of SLE, other inflammatory/autoimmune diseases or cancer. The demographic and clinical features were collected from the medical records or by questionnaire and reviewed by experienced physicians. The study was approved by the Medical Ethics Committee of Anhui Medical University. All participants were enrolled after informed consent had been obtained.

### SNP selection, genotyping and enzyme‐linked immunosorbent assay (ELISA)

We conducted a search for the *LEP* and *LEPR* gene single‐nucleotide polymorphisms (SNPs) with a minor allele frequency (MAF) ≥0.05 within the Han Chinese population (CHB) of Beijing, China, as listed in the international HapMap Project databank (http://hapmap.ncbi.nlm.nih.gov/cgi-perl/gbrowse/hapmap24_B36/; HapMap Data Rel 24/phasell Nov08, on NCBI B36 assembly, dbSNP b126). Then, linkage disequilibrium (LD) analysis with an *r* ^2^ threshold of 0.8 was performed with Haploview 4.2 software (Broad Institute, Cambridge, MA, USA) for tagging SNP selection. Under these criteria, six tag SNPs in *LEP* and 26 tag SNPs in *LEPR* were selected for further evaluation. We used the bioinformatics tools F‐SNP (http://com pbio.cs.queensu.ca/F‐SNP/) and SNP function prediction (http://snpinfo.niehs.nih.gov/snpinfo/snpfunc.htm) to assess the predicted functional effects of each tag SNP 16, 17. The test for functional SNP aimed to evaluate the potentially deleterious functional impact at the splicing, transcriptional, translational and post‐translational level. The basic information of these tag SNPs is shown in Table S1. In addition, the existing literature studies about the *LEP* and *LEPR* gene polymorphisms were reviewed. Finally, a total of four tag SNPs (rs11761556, rs12706832, rs2071045 and rs2167270) in *LEP* and nine tag SNPs [rs10749754, rs1137100, rs1137101, rs13306519, rs8179183 (rs1805094), rs1805096, rs3790434, rs3806318 and rs7518632] in *LEPR* were included for genotyping in our study cohort. The selected SNPs were genotyped in both case and control groups performed with improved multiple ligase detection reaction (iMLDR) genotyping assays, with technical support from the Center for Genetic & Genomic Analysis, Genesky Biotechnologies Inc., Shanghai.

Serum leptin level was determined by ELISA kits according to the manufacturer's instruction (R&D Systems, Inc. Minneapolis, MN, USA), and the results were expressed as nanogram per millilitre.

### Statistical analysis

Differences in genotype and allele frequencies between the two groups were analysed using chi‐square or Fisher's exact test. Comparisons of serum leptin level between different groups of clinical features and genotypes were conducted using nonparametric test. Odds ratios (*ORs*) and 95% confidence interval (*CIs*) were estimated by non‐conditional logistic regression analyses. All these statistical analyses were performed using SPSS 10.01 software (SPSS Inc., Chicago, IL, USA).

Hardy–Weinberg equilibrium (HWE) was evaluated in normal controls, and haplotype tests were performed by the SHEsis software (http://analysis.bio-x.cn/myAnalysis.php) 18. A two‐sided *P* value of <0.05 was considered as statistically significant. The Bonferroni correction was used for multiple testing.

## Results

In this study, we included a total of 633 SLE cases and 559 healthy controls. In patients, there were 60 males and 573 females with a mean age of 39.40 ± 12.74 years, while six males and 553 females in controls with a mean age of 42.92 ± 16.57 years. The clinical features of the patients are shown in Table 1. The main clinical manifestations were arthritis (46.52%), malar rash (36.47%), renal disorder (12.64%) and oral ulcers (11.99%) (Table 1). The observed genotype frequencies of all detected SNPs were distributed in compliance with the HWE in control groups (all *P* > 0.05).

**Table 1:** Demographic characteristics and clinical features of 633 patients with SLE

Characteristics	Patients with SLE (*n* = 633)
*Demographic characteristics*	
Age, year	39.4 ± 12.74
Male, *n* (%)	60 (9.48)
Female, *n* (%)	573 (90.52)
*Clinical manifestations*	
Malar rash, *n* (%)	225 (36.47)
Discoid rash, *n* (%)	65 (10.53)
Photosensitivity, *n* (%)	57 (9.24)
Oral ulcers, *n* (%)	74 (11.99)
Arthritis, *n* (%)	287 (46.52)
Pleurisy, *n* (%)	22 (3.57)
Pericarditis, *n* (%)	16 (2.59)
Renal disorder, *n* (%)	78 (12.64)
Neurological disorder, *n* (%)	22 (3.57)

*n*: number; SLE: systemic lupus erythematosus.

### Association of *LEP* and *LEPR* gene polymorphisms with risk of SLE

The result of allele frequency and genotype frequency of 13 SNPs in patients with SLE and controls are shown in Tables 2 & 3. There were no significant differences in allele and genotype distribution between patients with SLE and controls in all of these SNPs (all *P* > 0.05). We also evaluated the association of *LEP* and *LEPR* gene polymorphisms with SLE under dominant, recessive and additive model (Tables 2 & 3). Consistently, none of these polymorphisms achieved a significant difference between cases and controls (all *P* > 0.05).

**Table 2:** Genotype frequencies of *LEP* SNPs in patients with SLE and healthy controls

SNP	Analysed model		SLE (*N* = 633)	Control (*N* = 559)	*P* value^a^
rs11761556	Genetypes	AA	337	305	0.962
		CA	249	211	0.740
		CC	47	43	
	Additive model	AA	337	305	0.962
		CC	47	43
rs12706832	Genetypes	GG	347	317	0.924
		GA	249	209	0.813
		AA	37	33	
	Additive model	GG	347	317	0.924
		AA	37	33	
rs2071045	Genetypes	CC	191	187	0.774
		CT	336	263	0.087
		TT	106	109	
	Additive model	CC	191	187	0.774
		TT	106	109	
rs2167270	Genetypes	GG	391	369	0.727
		GA	215	167	0.760
		AA	27	23	
	Additive model	GG	391	369	0.726
		AA	27	23	

*N*: number; SNP: single‐nucleotide polymorphism; OR: odds ratio; CI: confidence interval.

a The *P* values are not corrected for multiple testings, Bonferroni corrected *P* = 0.0167.

**Table 3:** Genotype frequencies of *LEPR* SNPs in patients with SLE and healthy control

SNP	Analysed model		SLE (*N* = 633)	Control (*N* = 559)	*P* value^a^
rs10749754	Genetypes	AA	468	415	0.801
		GA	156	135	0.766
		GG	9	9	
	Additive model	AA	468	415	0.801
		GG	9	9	
rs1137100	Genetypes	GG	440	385	0.095
		GA	165	160	0.055
		AA	28	14	
	Additive model	GG	440	385	0.091
		AA	28	14	
rs1137101	Genetypes	GG	478	427	0.987
		GA	145	123	0.901
		AA	10	9	
	Additive model	GG	478	427	0.987
		AA	10	9	
rs13306519	Genetypes	CC	426	393	0.106
		CG	171	145	0.208
		GG	36	21	
	Additive model	CC	426	393	0.103
		GG	36	21	
rs8179183	Genetypes	CC	579	502	0.283
		GC	53	54	0.356
		GG	1	3	
	Additive model	CC	579	502	0.343^b^
		GG	1	3	
rs1805096	Genetypes	AA	480	437	0.508
		GA	145	112	0.327
		GG	8	10	
	Additive model	AA	480	437	0.506
		GG	8	10	
rs3790434	Genetypes	CC	446	415	0.235
		CT	178	130	0.088
		TT	9	14	
	Additive model	CC	446	415	0.230
		TT	9	14	
rs3806318	Genetypes	AA	512	448	0.262
		GA	112	107	0.214
		GG	9	4	
	Additive model	AA	512	448	0.279^b^
		GG	9	4	
rs7518632	Genetypes	AA	403	352	0.324
		CA	198	186	0.230
		CC	32	21	
	Additive model	AA	403	352	0.323
		CC	32	21	

*N*: number; SNP: single‐nucleotide polymorphism; OR: odds ratio; CI: confidence interval.

a The *P* values are not corrected for multiple testings, Bonferroni corrected *P* = 0.0167.

b Calculated by Fisher's exact test (exact *P* value).

### Association of *LEP* and *LEPR* gene polymorphisms with clinical features in patients with SLE

To examine the potential genetic association between leptin‐related gene polymorphisms and specific clinical features in SLE, we conducted a case‐only analysis and summarized the results in Tables 4 & 5. In *LEP*, the TT genotype and T allele frequencies of the rs2071045 polymorphism were significantly higher in patients with pericarditis compared with patients without this feature (*P* = 0.012, *P* = 0.011, respectively). The A allele of the rs11761556 was significantly higher in patients with malar rash (*P* = 0.044). However, no significant associations between other SNPs in *LEP* and clinical features of SLE were observed. In *LEPR*, the GA/GG genotype and G allele frequencies of the rs3806318 polymorphism achieved significant difference between patients with and without photosensitivity (*P* = 0.013, *P* = 0.008, respectively). In addition, the GA/AA genotype and A allele of rs1137100 also demonstrated an increased risk of photosensitivity (*P* = 0.043, *P* = 0.018, respectively) in patients with SLE. No significant associations of other SNPs in *LEPR* with clinical features were found. Furthermore, there was no significant difference in the genotype distribution between patients with SLEDAI >10 and patients with SLEDAI ≤10 (Tables 4 & 5).

**Table 4:** The positive findings of association between genotype frequencies in *LEP* and clinical characteristics

Gene (SNP)	Allele	Clinical features	Group	Genetypes *n* (%)			*P* value^a^
	(M/m)			MM	Mm	mm	
rs2071045	C/T	Pericarditis	Positive	2	7	7	**0.012**
			Negative	181	322	98	

*n*: number; SNP: single‐nucleotide polymorphism; M: major alleles; m: minor alleles; SLEDAI: systemic lupus erythematosus disease activity index.

a The *P* values are not corrected for multiple testings, Bonferroni corrected *P* = 0.0167. Bold value means *P*<0.05.

**Table 5:** The positive findings of association between genotype frequencies in *LEPR* and clinical characteristics

Gene (SNP)	Allele	Clinical features	Group	Genetypes *n* (%)			*P* value^a^
	(M/m)			MM	Mm	mm	
rs1137100	G/A	Photosensitivity	Positive	34	17	6	**0.043**
			Negative	396	142	22	
rs3806318	A/G	Photosensitivity	Positive	40	14	3	**0.013**
			Negative	458	96	6	
		Arthritis	Positive	237	43	7	**0.045**
			Negative	261	67	2	
		SLEDAI	More active (SLEDAI >10)	33	3	2	**0.020** b
			Less active (SLEDAI≤10)	37	16	1	

*n*: number; SNP: single‐nucleotide polymorphism; M: major alleles; m: minor alleles; SLEDAI: systemic lupus erythematosus disease activity index.

a The *P* values are not corrected for multiple testings, Bonferroni corrected *P* = 0.0167. Bold value means *P*<0.05.

b Calculated by Fisher's exact test (exact *P* value).

### Association of serum leptin levels with *LEP* and *LEPR* genotypes in patients with SLE

We examined the associations between serum leptin levels with different *LEP* and *LEPR* genotypes (Table 6). However, no significant differences in serum leptin levels were observed between patients with different *LEP* or *LEPR* genotypes.

**Table 6:** Association of serum leptin levels with genotype in *LEP* and *LEPR*

SNP	Genetypes	Number	Serum leptin level	*P* value
			M (P_25_, P_75_)	
rs11761556	AA	46	6.43 (3.75, 19.60)	0.838
	CA	37	6.34 (3.66, 18.54)	
	CC	8	8.87 (3.03, 13.14)	
rs12706832	GG	49	7.12 (3.94, 19.29)	0.334
	GA	36	5.75 (3.35, 16.53)	
	AA	6	12.53 (5.60, 21.25)	
rs2071045	CC	30	7.14 (3.59, 19.56)	0.928
	CT	47	5.86 (4.07, 20.36)	
	TT	14	8.87 (3.41, 16.45)	
rs2167270	GG	57	7.12 (3.73, 20.02)	0.428
	GA	31	5.85 (3.38,14.66)	
	AA	3	13.49 (6.17,18.98)	
rs10749754	AA	69	6.34 (3.57, 18.39)	0.832
	GA	18	5.53 (4.32, 19.51)	
	GG	4	14.27 (4.20, 20.89)	
rs1137100	GG	67	6.34 (3.61, 19.17)	0.454
	GA	18	11.26 (4.89, 19.51)	
	AA	6	5.13 (2.38, 15.95)	
rs1137101	GG	72	6.41 (3.63, 18.69)	0.955
	GA	16	5.47 (4.15, 20.08)	
	AA	3	12.52 (1.43, 16.02)	
rs13306519	CC	63	8.21 (4.11, 19.17)	0.186
	CG	21	5.05 (3.44, 12.46)	
	GG	7	16.70 (5.29, 22.51)	
rs8179183	CC	81	7.12 (3.70, 19.29)	0.113
	GC	10	4.40 (3.85, 7.29)	
	GG	0	0	
rs1805096	AA	73	6.26 (3.74, 18.39)	0.272
	GA	17	12.52 (4.05, 19.80)	
	GG	1	1.43	
rs3790434	CC	61	6.34 (3.57,19.08)	0.647
	CT	28	8.21 (4.12,18.96)	
	TT	2	4.68 (4.40,4.95)	
rs3806318	AA	69	6.48 (3.92,19.85)	0.396
	GA	19	10.81 (3.52,15.92)	
	GG	3	3.69 (3.61,5.21)	
rs7518632	AA	65	6.48 (3.66,19.08)	0.632
	CA	17	10.96 (4.18,19.80)	
	CC	9	5.54 (2.95,14.27)	

SNP: single‐nucleotide polymorphism; M: median.

### Haplotype analyses

Five main haplotypes (AATC, GATC, GGCA, GGCC and GGTA) for *LEP* gene and five main haplotypes (ACACGACGC, ACGCAGCAA, ACGGAGCAA, ATGCAGCAA and GCGCAGCAA) for *LEPR* gene were identified using SHEsis software (Tables 7 & 8). The results revealed that the haplotypes ACGCAGCAA and ATGCAGCAA were significantly associated with SLE, and the ACGCAGCAA appeared to be a significant protective haplotype (*P* = 0.003, OR = 0.745, 95% CI: 0.615–0.903; *P* = 0.038, OR = 1.390, 95% CI: 1.018–1.897).

**Table 7:** Haplotype analysis of four SNPs in *LEP* gene in patients with SLE and controls

Haplotype	Case [*n* (%)]	Control [*n* (%)]	χ^2^	*P* value	OR (95% CI)
rs2167270‐ rs12706832‐ rs2071045‐ rs11761556					
AATC	223.98 (17.7)	178.29 (15.9)	1.308	0.253	0.881 (0.710, 1.094)
GATC	51.11 (4.0)	55.50 (5.0)	1.193	0.275	1.242 (0.841, 1.833)
GGCA	646.32 (51.1)	577.65 (51.7)	0.089	0.765	1.025 (0.870, 1.209)
GGCC	64.28 (5.1)	57.97 (5.2)	0.014	0.906	1.022 (0.710, 1.472)
GGTA	226.09 (17.9)	201.06 (18.0)	0.006	0.939	1.008 (0.817, 1.245)

Total χ^2^ = 2.274, *P* = 0.069.

All the haplotypes with a frequency <0.03 were ignored in the analysis.

**Table 8:** Haplotype analysis of four SNPs in *LEPR* gene in patients with SLE and controls

Haplotype	Case [*n* (%)]	Control [*n* (%)]	χ^2^	*P* value	OR (95% CI)
rs3806318‐ rs3790434‐ rs1137100‐ rs13306519‐ rs10749754‐ rs1137101‐ rs8179183‐ rs1805096‐ rs7518632					
ACACGACGC	65.57 (5.2)	44.72 (4.0)	1.914	0.167	1.318 (0.891, 1.949)
ACGCAGCAA	587.70 (46.4)	577.78 (51.7)	9.038	**0.003**	0.745 (0.615, 0.903)
ACGGAGCAA	158.79 (12.5)	121.20 (10.8)	1.754	0.185	1.189 (0.920, 1.537)
ATGCAGCAA	110.74 (8.7)	72.77 (6.5)	4.327	**0.038**	1.390 (1.018, 1.897)
GCGCAGCAA	49.31 (3.9)	42.96 (3.8)	0.005	0.942	1.016 (0.668, 1.545)

Total χ^2^ = 10.469, *P* = 0.033. Bold value means *P*<0.05.

All the haplotype with a frequency <0.03 were ignored in the analysis.

## Discussion

Systemic lupus erythematosus is a severe autoimmune disease with multiple serological alterations and affecting various organs. Although the aetiology is still unclear, it is generally acknowledged that this disease has complex genetic and environmental backgrounds. Recent studies have shown that leptin levels are elevated in patients with SLE and involved in the pathogenesis of this disease 19, 20, 21, 22. However, some other groups have demonstrated lower or unchanged circulating leptin levels in patients with SLE compared to healthy controls 23, 24. Our recent work, a meta‐analysis, also found no significant difference in plasma/serum leptin level between patients with SLE and normal controls 25. Similar studies have also been conducted among various autoimmune diseases such as rheumatoid arthritis (RA) and osteoarthritis (OA) 26, 27, 28. A recent study showed that serum leptin level and serum leptin/leptin receptor ratio imbalance were positively correlated with anticyclic citrullinated peptide antibodies in RA 29 and might act as a predictor for disease activity 30. Therefore, *LEP* and *LEPR* gene polymorphisms might affect its expression and activity, and thereby involved in SLE pathogenesis.

In the current study, we investigated the association of *LEP* and *LEPR* single‐nucleotide polymorphisms with susceptibility to SLE in a Chinese population. However, we failed to detect any significant association between these SNPs and SLE risk. This result is inconsistent with a previous study by Afroze *et al*. They firstly reported an association between *LEPR Q223R* polymorphisms and risk of SLE in 100 Kashmiri individuals 10. They found that the carriers of variant genotype (A/G + G/G) or G allele were at increased risk of SLE and the different genotypes of *LEPR Q223R* might be involved in the development of different clinical manifestations associated with SLE. However, the sample size of this study is relatively small to get a powerful conclusion. Another study by Zhao *et al*. 11 also analysed the association of leptin pathway‐related gene polymorphisms with SLE risk in four different ancestral groups. Then, they conducted a trans‐ancestry meta‐analysis across four ancestral groups to elevate the overall effect among these SNPs. Their results suggested that although several SNPs showed weak associations, these associations did not remain significant after correction for multiple testing. This result was similar with our findings. We also examined the potential associations of *LEP* and *LEPR* gene polymorphisms with specific clinical characteristics in patients with SLE. In *LEP*, none of the SNPs were associated with any clinical characteristics except the rs2071045 polymorphism. The TT genotype and T allele frequencies of the rs2071045 were significantly increased in patients with pericarditis, suggesting that the T allele of rs2071045 in SLE might elevate the risk of pericarditis. In *LEPR*, we found positive association between the GA/GG genotype and G allele frequencies of the rs3806318 polymorphism and the risk of photosensitivity in patients with SLE. Our results also demonstrated that rs1137100 might increase the risk of skin photosensitivity in SLE; however, the differences did not reach the statistical significance after correction for multiple testing.

Previous studies have also evaluated the role of *LEP* and *LEPR* genes in multiple autoimmune diseases; however, the results are controversial 31, 32, 33, 34. A recent study demonstrated that the genotype and allele frequencies of the *LEP* rs2167270 gene polymorphism had no association with the risk of RA 31. However, Farrokhi *et al*. revealed a significant role of *LEP G‐2548‐A* and *LEPR Q223R* polymorphisms in the risk of multiple sclerosis and its severity 32. Furthermore, two recent studies showed that genetic polymorphisms of the leptin gene might influence lung function via Notch and JAK/STAT3 signalling pathway 34, 35. Thus, *LEP* and *LEPR* gene polymorphisms might influence the production and activity of inflammatory cytokines and thereby play a role in the development of various autoimmune diseases.

However, one limitation of our study should be acknowledged. The sample size for analysing the serum leptin level in patients with SLE may not be sufficient to get a solid conclusion. Therefore, the findings should be interpreted with caution; further studies with larger sample size are needed to confirm these results.

In conclusion, *LEP* and *LEPR* gene polymorphisms are not associated with genetic susceptibility to SLE in the Chinese population. However, some SNPs are associated with specific clinical phenotype of SLE, such as skin rash, pericarditis and arthritis. Compared with previous similar studies, some of these contradictions may be due to studies with different ethnic background, sample size, pathogenesis and patients with different disease activity, duration and treatment. Therefore, further studies based on larger sample size in different populations are required to confirm this result, and the gene–gene or genes–environment interaction should be taken into consideration.

## Conflict of interest

The authors confirm that there are no conflict of interest.

## Supporting information


**Table S1** Characteristics of the 32 Tag SNPs

**Table S2** Genotype and allele frequencies of *LEP* SNPs in SLE patients and health controls

**Table S3** Genotype and allele frequencies of *LEPR* SNPs in SLE patients and health controls

**Table S4** Association of clinical characteristics with genotype and allele frequencies in *LEP*

**Table S5** Association of clinical characteristics with genotype and allele frequencies in *LEPR*
