# Supplementary material for: Association of leptin and leptin receptor gene polymorphisms with systemic lupus erythematosus in a Chinese population
Source: J Cell Mol Med. 2017 Feb 28;21(9):1732–41. doi: 10.1111/jcmm.13093 (PMC5571531; doi:10.1111/jcmm.13093)
Supplement: Supplementary file 1 — Table S1 Characteristics of the 32 Tag SNPs [file JCMM-21-1732-s001.doc]

**Table S1** Characteristics of the 32 Tag SNPs

| rs_num | Chromosome | Position | Allele | GeneName | CHB_Hapmap | Functional Prediction | | | | | | | | | | | | | |
| --- | --- | --- | --- | --- | --- | --- | --- | --- | --- | --- | --- | --- | --- | --- | --- | --- | --- | --- | --- |
|  |  |  |  |  |  | FS score | TFBS | Splicing (site) | Splicing (ESE or ESS) | Splicing (abolish domain) | miRNA (miRanda) | miRNA (Sanger) | nsSNP | Stop Codon | Polyphen | SNPs3D (svm profile) | SNPs3D (svm structure) | RegPotential | Conservation |
| rs11760956 | 7 | 127678323 | A/G | LEP | 0.191 | -- | -- | -- | -- | -- | -- | -- | -- | -- | -- | -- | -- | 0 | 0 |
| rs12706832 | 7 | 127674375 | A/G | LEP | 0.223 | 0.176 | -- | -- | -- | -- | -- | -- | -- | -- | -- | -- | -- | 0 | 0.002 |
| rs2071045 | 7 | 127680216 | C/T | LEP | 0.423 | 0.208 | -- | -- | -- | -- | -- | -- | -- | -- | -- | -- | -- | NA | 0.007 |
| rs2167270 | 7 | 127668585 | A/G | LEP | 0.175 | 0.176 | Y | -- | Y | -- | -- | -- | -- | -- | -- | -- | -- | 0.412361 | 0.002 |
| rs11761556 | 7 | 127684305 | A/C | LEP | 0.259 | 0.176 | -- | -- | -- | -- | Y | -- | -- | -- | -- | -- | -- | 0 | 0 |
| rs7799039 | 7 | 127666019 | A/G | LEP | 0.202 | No functional information | Y | -- | -- | -- | -- | -- | -- | -- | -- | -- | -- | NA | 0 |
| rs6657632 | 1 | 65811254 | A/G | LEPR | 0.153 | 0.242 | -- | -- | -- | -- | -- | -- | -- | -- | -- | -- | -- | NA | 0 |
| rs4655528 | 1 | 65822077 | A/G | LEPR | 0.111 | 0.176 | -- | -- | -- | -- | -- | -- | -- | -- | -- | -- | -- | 0 | 0.018 |
| rs2104564 | 1 | 65742018 | C/T | LEPR | 0.122 | 0.208 | -- | -- | -- | -- | -- | -- | -- | -- | -- | -- | -- | 0 | 0 |
| rs1475398 | 1 | 65755845 | C/G | LEPR | 0.114 | 0 | -- | -- | -- | -- | -- | -- | -- | -- | -- | -- | -- | 0 | 0.011 |
| rs10736402 | 1 | 65830588 | C/T | LEPR | 0.136 | 0.176 | -- | -- | -- | -- | -- | -- | -- | -- | -- | -- | -- | 0 | 0.001 |
| rs1137101 | 1 | 65831101 | A/G | LEPR | 0.147 | 0.291 | -- | -- | Y | -- | -- | -- | Y | -- | possibly damaging | -- | -- | 0.006091 | 0.986 |
| rs7534511 | 1 | 65667718 | A/G | LEPR | 0.114 | 0.242 | -- | -- | -- | -- | -- | -- | -- | -- | -- | -- | -- | 0 | 0 |
| rs1046011 | 1 | 65671584 | T/C | LEPR | 0.135 | 0.065 | -- | -- | -- | -- | -- | -- | -- | -- | -- | -- | -- | 0 | 0 |
| rs1805096 | 1 | 65874845 | G/A | LEPR | 0.164 | 0.5 | -- | -- | -- | -- | -- | -- | -- | -- | -- | -- | -- | 0.111114 | 0 |
| rs4655557 | 1 | 65853375 | C/T | LEPR | 0.113 | 0.065 | -- | -- | -- | -- | -- | -- | -- | -- | -- | -- | -- | 0 | 0 |
| rs4655556 | 1 | 65853151 | A/G | LEPR | 0.117 | 0 | -- | -- | -- | -- | -- | -- | -- | -- | -- | -- | -- | 0 | 0 |
| rs7518632 | 1 | 65875938 | A/C | LEPR | 0.107 | 0.268 | -- | -- | -- | -- | -- | -- | -- | -- | -- | -- | -- | 0.068104 | 0.001 |
| rs6657868 | 1 | 65686295 | A/G | LEPR | 0.113 | 0.208 | -- | -- | -- | -- | -- | -- | -- | -- | -- | -- | -- | NA | 0 |
| rs3790434 | 1 | 65659036 | T/C | LEPR | 0.146 | 0 | Y | -- | -- | -- | -- | -- | -- | -- | -- | -- | -- | 0.259083 | 0 |
| rs7555955 | 1 | 65710067 | A/G | LEPR | 0.106 | 0 | -- | -- | -- | -- | -- | -- | -- | -- | -- | -- | -- | 0 | 0.001 |
| rs12029311 | 1 | 65755938 | A/G | LEPR | 0.153 | 0.176 | -- | -- | -- | -- | -- | -- | -- | -- | -- | -- | -- | 0 | 0.001 |
| rs7519977 | 1 | 65806084 | A/G | LEPR | 0.167 | 0.242 | -- | -- | -- | -- | -- | -- | -- | -- | -- | -- | -- | NA | 0 |
| rs1137100 | 1 | 65809029 | A/G | LEPR | 0.146 | 0.533 | -- | -- | Y | -- | -- | -- | Y | -- | benign | -- | -- | 0.06116 | 0.993 |
| rs12037879 | 1 | 65715295 | A/G | LEPR | 0.144 | 0.242 | -- | -- | -- | -- | -- | -- | -- | -- | -- | -- | -- | 0 | 0 |
| rs1782763 | 1 | 65780488 | C/T | LEPR | 0.111 | 0.242 | -- | -- | -- | -- | -- | -- | -- | -- | -- | -- | -- | 0 | 0.026 |
| rs10889568 | 1 | 65832033 | C/T | LEPR | 0.111 | 0.176 | -- | -- | -- | -- | -- | -- | -- | -- | -- | -- | -- | 0 | 0.013 |
| rs13306519 | 1 | 65810517 | C/G | LEPR | 0.179 | 0.242 | -- | -- | -- | -- | -- | -- | -- | -- | -- | -- | -- | 0.273101 | 0.083 |
| rs9436740 | 1 | 65664489 | A/T | LEPR | 0.179 | 0.242 | -- | -- | -- | -- | -- | -- | -- | -- | -- | -- | -- | NA | 0 |
| rs10749754 | 1 | 65827228 | A/G | LEPR | 0.114 | 0.208 | -- | -- | -- | -- | -- | -- | -- | -- | -- | -- | -- | 0 | 0.049 |
| rs3806318 | 1 | 65657945 | A/G | LEPR | 0.125 | 0.242 | Y | -- | -- | -- | -- | -- | -- | -- | -- | -- | -- | 0 | 0 |
| rs8179183 | 1 | 65848540 | C/G | LEPR | 0.043 | 0.533 | -- | -- | Y | -- | -- | -- | Y | -- | possibly damaging | -- | -- | 0.078724 | 0.958 |

CHB, Han Chinese in Beijing, China
