# Supplementary material for: Association of leptin and leptin receptor gene polymorphisms with systemic lupus erythematosus in a Chinese population
Source: J Cell Mol Med. 2017 Feb 28;21(9):1732–41. doi: 10.1111/jcmm.13093 (PMC5571531; doi:10.1111/jcmm.13093)
Supplement: Supplementary file 2 — Table S2 Genotype and allele frequencies of LEP SNPs in SLE patients and health controls [file JCMM-21-1732-s002.doc]

**Table S2** Genotype and allele frequencies of *LEP* SNPs in SLE patients and health controls

| SNP | Analyze model | | SLE (N = 633) n (%) | Control (N = 559) n (%) | χ2 | *P* value* | OR (95% CI) |
| --- | --- | --- | --- | --- | --- | --- | --- |
| rs11761556 | Genetypes | AA | 337 (53.3) | 305 (54.6) | 0.002 | 0.962 | 0.989 (0.636, 1.539) |
|  |  | CA | 249 (39.3) | 211 (37.7) | 0.110 | 0.740 | 0.926 (0.589, 1.456) |
|  |  | CC | 47 (7.4) | 43 (7.7) | Reference | | |
|  | Alleles | A | 923 (72.9) | 821 (73.4) | 0.084 | 0.772 | 0.973 (0.812, 1.167) |
|  |  | C | 343 (27.1) | 297 (26.6) | Reference | | |
|  | Dominant model | AA | 337 (53.2) | 305 (54.6) | 0.209 | 0.647 | 0.948 (0.755, 1.191) |
|  |  | CC+CA | 296 (46.8) | 254 (45.4) | Reference | | |
|  | Recessive model | CA+AA | 586 (92.6) | 516 (92.3) | 0.030 | 0.862 | 1.039 (0.676, 1.598) |
|  |  | CC | 47 (7.4) | 43 (7.7) | Reference | | |
|  | Additive model | AA | 337 (87.8) | 305 (87.6) | 0.002 | 0.962 | 1.011 (0.650, 1.572) |
|  |  | CC | 47 (12.2) | 43 (12.4) | Reference | | |
| rs12706832 | Genetypes | GG | 347 (54.8) | 317 (56.7) | 0.009 | 0.924 | 1.024 (0.625, 1.678) |
|  |  | GA | 249 (39.3) | 209 (37.4) | 0.056 | 0.813 | 0.922 (0.569, 1.558) |
|  |  | AA | 37 (5.9) | 33 (5.9) | Reference | | |
|  | Alleles | G | 943 (74.5) | 843 (75.4) | 0.265 | 0.607 | 1.050 (0.872, 1.264) |
|  |  | A | 343 (25.5) | 275 (24.6) | Reference | | |
|  | Dominant model | GG | 347 (54.8) | 317 (56.7) | 0.430 | 0.512 | 1.080 (0.859, 1.358) |
|  |  | AA+GA | 286 (45.2) | 242 (43.3) | Reference | | |
|  | Recessive model | GA+GG | 596 (94.2) | 526 (94.1) | 0.002 | 0.966 | 0.990 (0.610, 1.605) |
|  |  | AA | 37 (5.8) | 33 (5.9) | Reference | | |
|  | Additive model | GG | 347 (90.4) | 317 (90.6) | 0.009 | 0.924 | 1.024 (0.625, 1.678) |
|  |  | AA | 37 (9.6) | 33 (9.4) | Reference | | |
| rs2071045 | Genetypes | CC | 191 (30.2) | 187 (33.5) | 0.082 | 0.774 | 0.952 (0.681, 1.331) |
|  |  | CT | 336 (53.1) | 263 (47.0) | 2.933 | 0.087 | 0.761 (0.557, 1.040) |
|  |  | TT | 106 (16.7) | 109 (19.5) | Reference | | |
|  | Alleles | C | 718 (56.7) | 637 (57.0) | 0.017 | 0.897 | 0.989 (0.841, 1.164) |
|  |  | T | 548 (43.3) | 481 (43.0) | Reference | | |
|  | Dominant model | CC | 191 (30.2) | 187 (33.5) | 1.474 | 0.225 | 0.860 (0.673, 1.097) |
|  |  | TT+CT | 442 (69.8) | 372 (66.5) | Reference | | |
|  | Recessive model | CT+CC | 527 (83.3) | 450 (80.5) | 1.522 | 0.217 | 1.204 (0.896, 1.618) |
|  |  | TT | 106 (16.7) | 109 (19.5) | Reference | | |
|  | Additive model | CC | 191 (64.3) | 187 (63.2) | 0.083 | 0.774 | 1.050 (0.751, 1.468) |
|  |  | TT | 106 (35.7) | 109 (36.8) | Reference | | |
| rs2167270 | Genetypes | GG | 391 (61.8) | 369 (66.0) | 0.122 | 0.727 | 1.108 (0.624, 1.967) |
|  |  | GA | 215 (34.0) | 167 (29.9) | 0.093 | 0.760 | 0.912 (0.505, 1.648) |
|  |  | AA | 27 (4.3) | 23 (4.1) | Reference | | |
|  | Alleles | G | 997 (78.8) | 905 (80.9) | 1.775 | 0.183 | 1.146 (0.938, 1.402) |
|  |  | A | 269 (21.2) | 213 (19.1) | Reference | | |
|  | Dominant model | GG | 391 (61.8) | 369 (66.0) | 2.311 | 0.128 | 1.202 (0.948, 1.524) |
|  |  | AA+GA | 242 (38.2) | 190 (34.0) | Reference | | |
|  | Recessive model | GA+GG | 606 (95.7) | 536 (95.9) | 0.017 | 0.897 | 1.038 (0.588, 1.833) |
|  |  | AA | 27 (4.3) | 23 (4.1) | Reference | | |
|  | Additive model | GG | 391 (93.5) | 369 (94.1) | 0.122 | 0.726 | 1.108 (0.624, 1.967) |
|  |  | AA | 27 (6.5) | 23 (5.9) | Reference | | |

N, number; SNP, single-nucleotide polymorphism; OR, odds ratio; CI, confidence interval.

* The *p* values are not corrected for multiple testings, Bonferroni corrected *p* = 0.0167.
