# Supplementary material for: Association of leptin and leptin receptor gene polymorphisms with systemic lupus erythematosus in a Chinese population
Source: J Cell Mol Med. 2017 Feb 28;21(9):1732–41. doi: 10.1111/jcmm.13093 (PMC5571531; doi:10.1111/jcmm.13093)
Supplement: Supplementary file 3 — Table S3 Genotype and allele frequencies of LEPR SNPs in SLE patients and health controls [file JCMM-21-1732-s003.doc]

**Table S3** Genotype and allele frequencies of *LEPR* SNPs in SLE patients and health controls

| SNP | Analyze model | | SLE (N = 633) n (%) | Control (N = 559) n (%) | χ2 | *P* value* | OR (95% CI) |
| --- | --- | --- | --- | --- | --- | --- | --- |
| rs10749754 | Genetypes | AA | 468 (73.9) | 415 (74.2) | 0.064 | 0.801 | 0.887 (0.349, 2.255) |
|  |  | GA | 156 (24.7) | 135 (24.2) | 0.089 | 0.766 | 0.543 (0.334, 2.243) |
|  |  | GG | 9 (1.4) | 9 (1.6) | Reference | | |
|  | Alleles | A | 1092 (86.3) | 965 (86.3) | 0.002 | 0.967 | 0.995 (0.788, 1.257) |
|  |  | G | 174 (13.7) | 153 (13.7) | Reference | | |
|  | Dominant model | AA | 468 (73.9) | 415 (74.2) | 0.014 | 0.904 | 0.984 (0.759, 1.276) |
|  |  | GG+GA | 165 (26.1) | 144 (25.8) | Reference | | |
|  | Recessive model | GA+AA | 624 (98.6) | 550 (98.4) | 0.071 | 0.790 | 1.135 (0.447, 2.878) |
|  |  | GG | 9 (1.4) | 9 (1.6) | Reference | | |
|  | Additive model | AA | 468 (98.1) | 415 (97.9) | 0.064 | 0.801 | 1.128 (0.443, 2.868) |
|  |  | GG | 9 (1.9) | 9 (2.1) | Reference | | |
| rs1137100 | Genetypes | GG | 440 (69.5) | 385 (68.9) | 2.796 | 0.095 | 1.750 (0.908, 3.372) |
|  |  | GA | 165 (26.1) | 160 (28.6) | 3.673 | 0.055 | 1.939 (0.985, 3.818) |
|  |  | AA | 28 (4.4) | 14 (2.5) | Reference | | |
|  | Alleles | G | 1045 (82.5) | 930 (83.2) | 0.172 | 0.679 | 1.046 (0.845, 1.295) |
|  |  | A | 221 (17.5) | 188 (16.8) | Reference | | |
|  | Dominant model | GG | 440 (69.5) | 385 (68.9) | 0.057 | 0.812 | 0.971 (0.759, 1.242) |
|  |  | AA+GA | 193 (30.5) | 174 (31.1) | Reference | | |
|  | Recessive model | GA+GG | 605 (95.6) | 545 (97.5) | 3.216 | 0.073 | 1.802 (0.939, 3.458) |
|  |  | AA | 28 (4.4) | 14 (2.5) | Reference | | |
|  | Additive model | GG | 440 (94.0) | 385 (96.5) | 2.860 | 0.091 | 1.750 (0.908, 3.372) |
|  |  | AA | 28 (6.0) | 14 (3.5) | Reference | | |
| rs1137101 | Genetypes | GG | 478 (75.5) | 427 (76.4) | 0.000 | 0.987 | 0.993 (0.400, 2.466) |
|  |  | GA | 145 (22.9) | 123 (22.0) | 0.015 | 0.901 | 0.943 (0.371, 2.394) |
|  |  | AA | 10 (1.6) | 9 (1.6) | Reference | | |
|  | Alleles | G | 1101 (87.0) | 977 (87.4) | 0.094 | 0.759 | 1.038 (0.816, 1.321) |
|  |  | A | 165 (13.0) | 141 (12.6) | Reference | | |
|  | Dominant model | GG | 478 (75.5) | 427 (76.4) | 0.124 | 0.725 | 1.049 (0.804, 1.369) |
|  |  | AA+GA | 155 (24.5) | 132 (23.6) | Reference | | |
|  | Recessive model | GA+GG | 623 (98.4) | 550 (98.4) | 0.002 | 0.967 | 0.981 (0.396, 2.432) |
|  |  | AA | 10 (1.6) | 9 (1.6) | Reference | | |
|  | Additive model | GG | 478 (98.0) | 427 (97.9) | 0.000 | 0.987 | 0.993 (0.400, 2.466) |
|  |  | AA | 10 (2.0) | 9 (2.1) | Reference | | |
| rs13306519 | Genetypes | CC | 426 (67.3) | 393 (70.3) | 2.617 | 0.106 | 1.581 (0.908, 2.756) |
|  |  | CG | 171 (27.0) | 145 (25.9) | 1.588 | 0.208 | 1.454 (0.812, 2.601) |
|  |  | GG | 36 (5.7) | 21 (3.8) | Reference | | |
|  | Alleles | C | 1023 (80.8) | 931 (83.3) | 2.446 | 0.118 | 0.846 (0.685, 1.044) |
|  |  | G | 243 (19.2) | 187 (16.7) | Reference | | |
|  | Dominant model | CC | 426 (67.3) | 393 (70.3) | 1.247 | 0.264 | 0.869 (0.680, 1.112) |
|  |  | GG+CG | 207 (32.7) | 166 (29.7) | Reference | | |
|  | Recessive model | CG+CC | 597 (94.3) | 538 (96.2) | 2.430 | 0.119 | 0.647 (0.373, 1.123) |
|  |  | GG | 36 (5.7) | 21 (3.8) | Reference | | |
|  | Additive model | CC | 426 (92.2) | 393 (94.9) | 2.655 | 0.103 | 0.632 (0.363, 1.102) |
|  |  | GG | 36 (7.8) | 21 (5.1) | Reference | | |
| rs8179183 | Genetypes | CC | 579 (91.5) | 502 (89.8) | 1.152 | 0.283 | 0.289 (0.030, 2.787) |
|  |  | GC | 53 (8.4) | 54 (9.7) | 0.851 | 0.356 | 0.340 (0.034, 3.369) |
|  |  | GG | 1 (0.2) | 3 (0.5) | Reference | | |
|  | Alleles | C | 1211 (95.7) | 1058 (94.6) | 1.352 | 0.245 | 1.249 (0.858, 1.817) |
|  |  | G | 55 (4.3) | 60 (5.4) | Reference | | |
|  | Dominant model | CC | 579 (91.5) | 502 (89.8) | 0.976 | 0.323 | 1.217 (0.824, 1.800) |
|  |  | GG+GC | 54 (8.5) | 57 (10.2) | Reference | | |
|  | Recessive model | GC+CC | 632 (99.8) | 556 (99.5) | --- | 0.346^a^ | 3.410 (0.354, 32.877) |
|  |  | GG | 1 (0.2) | 3 (0.5) | Reference | | |
|  | Additive model | CC | 579 (99.8) | 502 (99.4) | --- | 0.343^a^ | 3.460 (0.359, 33.369) |
|  |  | GG | 1 (0.2) | 3 (0.6) | Reference | | |
| rs1805096 | Genetypes | AA | 480 (75.8) | 437 (78.2) | 0.438 | 0.508 | 0.728 (0.285, 1.862) |
|  |  | GA | 145 (22.9) | 112 (20.0) | 0.962 | 0.327 | 0.618 (0.236, 1.617) |
|  |  | GG | 8 (1.3) | 10 (1.8) | Reference | | |
|  | Alleles | A | 1105 (87.3) | 986 (88.2) | 0.457 | 0.499 | 0.919 (0.719, 1.175) |
|  |  | G | 142 (12.7) | 132 (11.8) | Reference | | |
|  | Dominant model | AA | 480 (75.8) | 437 (78.2) | 0.920 | 0.337 | 0.876 (0.668, 1.148) |
|  |  | GG+GA | 153 (24.2) | 122 (21.8) | Reference | | |
|  | Recessive model | GA+AA | 625 (98.7) | 549 (98.2) | 0.550 | 0.458 | 1.423 (0.558, 3.631) |
|  |  | GG | 8 (1.3) | 10 (1.8) | Reference | | |
|  | Additive model | AA | 480 (98.4) | 437 (97.8) | 0.442 | 0.506 | 1.373 (0.537, 3.510) |
|  |  | GG | 8 (1.6) | 10 (2.2) | Reference | | |
| rs3790434 | Genetypes | CC | 446 (70.5) | 415 (74.2) | 1.411 | 0.235 | 0.598 (0.256, 1.397) |
|  |  | CT | 178 (28.1) | 130 (23.3) | 2.919 | 0.088 | 0.470 (0.197, 1.118) |
|  |  | TT | 9 (1.4) | 14 (2.5) | Reference | | |
|  | Alleles | C | 1070 (84.5) | 960 (85.9) | 0.855 | 0.355 | 0.898 (0.716, 1.127) |
|  |  | T | 196 (15.5) | 158 (14.1) | Reference | | |
|  | Dominant model | CC | 446 (70.5) | 415 (74.2) | 2.116 | 0.146 | 0.828 (0.641, 1.068) |
|  |  | TT+CT | 187 (29.5) | 144 (25.8) | Reference | | |
|  | Recessive model | CT+CC | 624 (98.6) | 545 (97.5) | 1.839 | 0.175 | 1.781 (0.765, 4.147) |
|  |  | TT | 9 (1.4) | 14 (2.5) | Reference | | |
|  | Additive model | CC | 446 (98.0) | 415 (96.7) | 1.440 | 0.230 | 1.672 (0.716, 3.903) |
|  |  | TT | 9 (2.0) | 14 (3.3) | Reference | | |
| rs3806318 | Genetypes | AA | 512 (80.9) | 448 (80.1) | 1.256 | 0.262 | 1.969 (0.602, 6.437) |
|  |  | GA | 112 (17.7) | 107 (19.1) | 1.544 | 0.214 | 2.150 (0.643, 7.189) |
|  |  | GG | 9 (1.4) | 4 (0.7) | Reference | | |
|  | Alleles | A | 1136 (89.7) | 1003 (89.7) | 0.000 | 0.989 | 1.002 (0.769, 1.306) |
|  |  | G | 130 (10.3) | 115 (10.3) | Reference | | |
|  | Dominant model | AA | 512 (80.9) | 448 (80.1) | 0.104 | 0.747 | 1.048 (0.787, 1.397) |
|  |  | GG+GA | 121 (19.1) | 111 (19.9) | Reference | | |
|  | Recessive model | GA+AA | 624 (98.6) | 555 (99.3) | --- | 0.276^a^ | 0.500 (0.153, 1.632) |
|  |  | GG | 9 (1.4) | 4 (0.7) | Reference | | |
|  | Additive model | AA | 512 (98.3) | 448 (99.1) | --- | 0.279^a^ | 0.508 (0.155, 1.661) |
|  |  | GG | 9 (1.7) | 4 (0.9) | Reference | | |
| rs7518632 | Genetypes | AA | 403 (63.7) | 352 (63.0) | 0.971 | 0.324 | 1.331 (0.754, 2.350) |
|  |  | CA | 198 (31.3) | 186 (33.3) | 1.441 | 0.230 | 1.431 (0.797, 2.571) |
|  |  | CC | 32 (5.0) | 21 (3.8) | Reference | | |
|  | Alleles | A | 1004 (79.3) | 890 (79.6) | 0.033 | 0.856 | 0.982 (0.804, 1.198) |
|  |  | C | 262 (20.7) | 228 (20.4) | Reference | | |
|  | Dominant model | AA | 403 (63.7) | 352 (63.0) | 0.062 | 0.804 | 1.030 (0.814, 1.305) |
|  |  | CC+CA | 230 (36.3) | 207 (37.0) | Reference | | |
|  | Recessive model | CA+AA | 601 (94.9) | 538 (96.2) | 1.178 | 0.278 | 0.733 (0.418, 1.287) |
|  |  | CC | 32 (5.1) | 21 (3.8) | Reference | | |
|  | Additive model | AA | 403 (92.6) | 352 (94.4) | 0.976 | 0.323 | 0.751 (0.425, 1.327) |
|  |  | CC | 32 (7.4) | 21 (5.6) | Reference | | |

N, number; SNP, single-nucleotide polymorphism; OR, odds ratio; CI, confidence interval.

* The *p* values are not corrected for multiple testings, Bonferroni corrected *p* = 0.0167; ^a^ Calculated by Fisher’ exact test (exact *p* value).
