# Supplementary material for: Association of leptin and leptin receptor gene polymorphisms with systemic lupus erythematosus in a Chinese population
Source: J Cell Mol Med. 2017 Feb 28;21(9):1732–41. doi: 10.1111/jcmm.13093 (PMC5571531; doi:10.1111/jcmm.13093)
Supplement: Supplementary file 4 — Table S4 Association of clinical characteristics with genotype and allele frequencies in LEP [file JCMM-21-1732-s004.doc]

**Table S4** Association of clinical characteristics with genotype and allele frequencies in *LEP*

| Gene (SNP) | Allele | Clinical features | Group | Genetypes n (%) | | | χ2 | *P* value* | Alleles n (%) | | χ2 | *P* value |
| --- | --- | --- | --- | --- | --- | --- | --- | --- | --- | --- | --- | --- |
|  | (M/m) |  |  | MM | Mm | mm |  |  | M | m |  |  |
| rs11761556 | A/C | Malar rash | Positive | 129 (57.3) | 85 (37.8) | 11 (4.9) | 4.521 | 0.104 | 343 (76.2) | 107 (23.8) | 4.067 | **0.044** |
|  |  |  | Negative | 199 (50.8) | 158 (40.3) | 35 (8.9) |  |  | 556 (70.9) | 228 (29.1) |  |  |
|  |  | Discoid rash | Positive | 35 (53.8) | 28 (43.1) | 2 (3.1) | 2.126 | 0.345 | 98 (75.4) | 32 (24.6) | 0.471 | 0.492 |
|  |  |  | Negative | 293 (53.1) | 215 (38.9) | 44 (8.0) |  |  | 801 (72.6) | 303 (27.4) |  |  |
|  |  | Photosensitivity | Positive | 27 (47.4) | 25 (43.9) | 5 (8.8) | 0.862 | 0.650 | 79 (69.3) | 35 (30.7) | 0.802 | 0.370 |
|  |  |  | Negative | 301 (53.8) | 218 (38.9) | 41 (7.3) |  |  | 820 (73.2) | 300 (26.8) |  |  |
|  |  | Oral ulcers | Positive | 39 (52.7) | 27 (36.5) | 8 (10.8) | 1.452 | 0.484 | 105 (70.9) | 43 (29.1) | 0.309 | 0.578 |
|  |  |  | Negative | 289 (53.2) | 216 (39.8) | 38 (7.0) |  |  | 794 (73.1) | 292 (26.9) |  |  |
|  |  | Arthritis | Positive | 156 (54.4) | 104 (36.2) | 27 (9.8) | 4.237 | 0.120 | 416 (72.5) | 158 (27.5) | 0.078 | 0.780 |
|  |  |  | Negative | 172 (52.1) | 139 (42.1) | 19 (5.8) |  |  | 483 (73.2) | 177 (26.8) |  |  |
|  |  | Pleurisy | Positive | 11 (50.0) | 10 (45.5) | 1 (4.5) | 0.515 | 0.773 | 32 (72.7) | 12 (27.3) | 0.000 | 0.985 |
|  |  |  | Negative | 317 (53.3) | 233 (39.2) | 45 (7.6) |  |  | 867 (72.9) | 323 (27.1) |  |  |
|  |  | Pericarditis | Positive | 7 (43.8) | 7 (43.8) | 2 (12.5) | 0.914 | 0.633 | 21 (65.6) | 11 (34.4) | 0.868 | 0.352 |
|  |  |  | Negative | 321 (53.4) | 236(39.3) | 44 (7.3) |  |  | 878 (73.0) | 324 (27.0) |  |  |
|  |  | Renal disorder | Positive | 37 (47.4) | 36 (46.2) | 5 (6.4) | 1.720 | 0.423 | 110 (70.5) | 46 (29.5) | 0.494 | 0.482 |
|  |  |  | Negative | 291 (54.0) | 207 (38.4) | 41 (7.6) |  |  | 789 (73.2) | 289 (26.8) |  |  |
|  |  | Neurological disorder | Positive | 15 (68.2) | 6 (27.3) | 1 (4.5) | 2.077 | 0.354 | 36 (81.8) | 8 (18.2) | 1.854 | 0.173 |
|  |  |  | Negative | 313 (52.6) | 237 (39.8) | 45 (7.6) |  |  | 863 (72.5) | 327 (27.5) |  |  |
|  |  | SLEDAI | More active (SLEDAI＞10) | 22 (57.9) | 14 (36.8) | 2 (5.3) | 1.648 | 0.484 | 58 (76.3) | 18 (23.7) | 1.655 | 0.198 |
|  |  |  | Less active (SLEDAI≤10) | 25 (46.3) | 23 (42.6) | 6 (11.1) |  |  | 73 (67.6) | 35 (32.4) |  |  |
| rs12706832 | G/A | Malar rash | Positive | 130 (57.8) | 86 (38.2) | 9 (4.0) | 3.025 | 0.220 | 346 (76.9) | 104 (23.1) | 2.318 | 0.128 |
|  |  |  | Negative | 208 (53.1) | 156 (39.8) | 28 (7.1) |  |  | 572 (73.0) | 212 (27.0) |  |  |
|  |  | Discoid rash | Positive | 32 (49.2) | 32 (49.2) | 1 (1.5) | 4.672 | 0.097 | 96 (73.8) | 34 (26.2) | 0.023 | 0.880 |
|  |  |  | Negative | 306 (55.4) | 210 (38.0) | 36 (6.6) |  |  | 822 (74.5) | 282 (25.5) |  |  |
|  |  | Photosensitivity | Positive | 27 (47.4) | 26 (38.0) | 4 (7.0) | 1.393 | 0.498 | 80 (70.2) | 34 (29.8) | 1.172 | 0.279 |
|  |  |  | Negative | 311 (55.5) | 216 (38.6) | 33 (5.9) |  |  | 838 (74.8) | 282 (25.2) |  |  |
|  |  | Oral ulcers | Positive | 38 (51.4) | 29 (39.2) | 7 (9.5) | 1.862 | 0.394 | 105 (70.9) | 43 (29.1) | 1.048 | 0.306 |
|  |  |  | Negative | 300 (55.2) | 213 (39.2) | 30 (5.5) |  |  | 813 (74.9) | 273 (25.1) |  |  |
|  |  | Arthritis | Positive | 164 (57.1) | 102 (35.5) | 21 (7.3) | 3.961 | 0.138 | 430 (74.9) | 144 (25.1) | 0.153 | 0.696 |
|  |  |  | Negative | 174 (52.7) | 140 (42.4) | 16 (4.8) |  |  | 488 (73.9) | 172 (26.1) |  |  |
|  |  | Pleurisy | Positive | 12 (54.5) | 9 (40.9) | 1 (4.5) | 0.097 | 0.953 | 33 (75.0) | 11 (25.0) | 0.009 | 0.925 |
|  |  |  | Negative | 326 (54.8) | 233 (39.2) | 36 (6.1) |  |  | 885 (74.4) | 305 (25.6) |  |  |
|  |  | Pericarditis | Positive | 7 (43.8) | 7 (43.8) | 2 (12.5) | 1.609 | 0.447 | 21 (65.6) | 11 (34.4) | 1.326 | 0.250 |
|  |  |  | Negative | 331 (55.1) | 235 (39.1) | 35 (5.8) |  |  | 897 (74.6) | 305 (25.4) |  |  |
|  |  | Renal disorder | Positive | 40 (51.3) | 33 (42.3) | 5 (6.4) | 0.442 | 0.802 | 113 (72.4) | 43 (27.6) | 0.359 | 0.549 |
|  |  |  | Negative | 298 (55.3) | 209 (38.8) | 32 (5.9) |  |  | 805 (74.7) | 273 (25.3) |  |  |
|  |  | Neurological disorder | Positive | 14 (63.6) | 7 (31.8) | 1 (4.5) | 0.726 | 0.696 | 35 (79.5) | 9 (20.5) | 0.636 | 0.425 |
|  |  |  | Negative | 324 (54.5) | 235 (39.5) | 36 (6.1) |  |  | 883 (74.2) | 307 (25.8) |  |  |
|  |  | SLEDAI | More active (SLEDAI＞10) | 24 (63.2) | 12 (31.6) | 2 (5.3) | --- | 0.330^a^ | 60 (78.9) | 16 (21.1) | 1.702 | 0.192 |
|  |  |  | Less active (SLEDAI≤10) | 26 (48.1) | 24 (44.4) | 4 (7.4) |  |  | 76 (70.4) | 32 (29.6) |  |  |
| rs2071045 | C/T | Malar rash | Positive | 70 (31.1) | 119 (52.9) | 36 (16.0) | 0.480 | 0.787 | 259 (57.6) | 191 (42.4) | 0.439 | 0.508 |
|  |  |  | Negative | 113 (28.8) | 210 (53.6) | 69 (17.6) |  |  | 436 (55.6) | 348 (44.4) |  |  |
|  |  | Discoid rash | Positive | 22 (33.8) | 29 (44.6) | 14 (21.5) | 2.335 | 0.302 | 73 (56.2) | 57 (43.8) | 0.002 | 0.968 |
|  |  |  | Negative | 161 (29.2) | 300 (54.3) | 91 (16.5) |  |  | 622 (56.3) | 482 (43.7) |  |  |
|  |  | Photosensitivity | Positive | 12 (21.1) | 33 (57.9) | 12 (21.1) | 2.416 | 0.299 | 57 (50.0) | 57 (50.0) | 2.040 | 0.153 |
|  |  |  | Negative | 171 (30.5) | 296 (52.9) | 93 (16.6) |  |  | 638 (57.0) | 482 (43.0) |  |  |
|  |  | Oral ulcers | Positive | 19 (25.7) | 42 (56.8) | 13 (17.6) | 0.651 | 0.722 | 80 (54.1) | 68 (45.9) | 0.351 | 0.553 |
|  |  |  | Negative | 164 (30.2) | 287 (52.9) | 92 (16.9) |  |  | 615 (56.6) | 471 (43.4) |  |  |
|  |  | Arthritis | Positive | 95 (33.1) | 141 (49.1) | 51 (17.8) | 4.091 | 0.129 | 331 (57.7) | 243 (42.3) | 0.789 | 0.374 |
|  |  |  | Negative | 88 (26.7) | 188 (57.0) | 54 (16.4) |  |  | 364 (55.2) | 296 (44.8) |  |  |
|  |  | Pleurisy | Positive | 6 (27.3) | 10 (45.5) | 6 (27.3) | 1.718 | 0.423 | 22 (50.0) | 22 (50.0) | 0.741 | 0.389 |
|  |  |  | Negative | 177 (29.7) | 319 (53.6) | 99 (16.6) |  |  | 673 (56.6) | 517 (43.4) |  |  |
|  |  | Pericarditis | Positive | 2 (12.5) | 7 (43.8) | 7 (43.8) | 8.811 | **0.012** | 11 (34.4) | 21 (65.6) | 6.432 | **0.011** |
|  |  |  | Negative | 181 (30.1) | 322 (53.6) | 98 (16.3) |  |  | 684 (56.9) | 518 (43.1) |  |  |
|  |  | Renal disorder | Positive | 23 (29.5) | 43 (55.1) | 12 (15.4) | 0.195 | 0.907 | 89 (57.1) | 67 (42.9) | 0.039 | 0.844 |
|  |  |  | Negative | 160 (29.7) | 286 (53.1) | 93 (17.3) |  |  | 606 (56.2) | 472 (43.8) |  |  |
|  |  | Neurological disorder | Positive | 7 (31.8) | 10 (45.5) | 5 (22.7) | 0.738 | 0.692 | 24 (54.5) | 20 (45.5) | 0.058 | 0.809 |
|  |  |  | Negative | 176 (29.6) | 319 (53.6) | 100 (16.8) | |  | 671 (56.4) | 519 (43.6) |  |  |
|  |  | SLEDAI | More active (SLEDAI＞10) | 12 (31.6) | 19 (50.0) | 7 (18.4) | 0.516 | 0.806 | 43 (56.6) | 33 (43.4) | 0.239 | 0.625 |
|  |  |  | Less active (SLEDAI≤10) | 18 (33.3) | 29 (53.7) | 7 (13.0) |  |  | 65 (60.2) | 43 (39.8) |  |  |
| rs2167270 | G/A | Malar rash | Positive | 147 (65.3) | 72 (32.0) | 6 (2.7) | 3.468 | 0.177 | 366 (81.3) | 84 (18.7) | 2.957 | 0.085 |
|  |  |  | Negative | 234 (59.7) | 137 (34.9) | 21 (5.4) |  |  | 605 (77.2) | 179 (22.8) |  |  |
|  |  | Discoid rash | Positive | 38 (58.5) | 27 (41.5) | 0 (0.00) | --- | 0.094^a^ | 103 (79.2) | 27 (20.8) | 0.026 | 0.873 |
|  |  |  | Negative | 343 (62.1) | 182 (33.0) | 27 (4.9) |  |  | 868 (78.6) | 236 (21.4) |  |  |
|  |  | Photosensitivity | Positive | 31 (54.4) | 22 (38.6) | 4 (7.0) | 1.967 | 0.374 | 84 (73.7) | 30 (26.3) | 1.875 | 0.171 |
|  |  |  | Negative | 350 (62.5) | 187 (33.4) | 23 (4.1) |  |  | 887 (79.2) | 233 (20.8) |  |  |
|  |  | Oral ulcers | Positive | 47 (63.5) | 23 (31.1) | 4 (5.4) | 0.440 | 0.803 | 117 (79.1) | 31 (20.9) | 0.013 | 0.908 |
|  |  |  | Negative | 334 (61.5) | 186 (34.3) | 23 (4.2) |  |  | 854 (78.6) | 232 (21.4) |  |  |
|  |  | Arthritis | Positive | 185 (64.5) | 89 (31.0) | 13 (4.5) | 1.965 | 0.374 | 459 (80.0) | 115 (20.0) | 1.045 | 0.307 |
|  |  |  | Negative | 196 (59.4) | 120 (36.4) | 14 (4.2) |  |  | 512 (77.6) | 148 (22.4) |  |  |
|  |  | Pleurisy | Positive | 16 (72.7) | 6 (27.3) | 0 (0.00) | --- | 0.399^a^ | 38 (86.4) | 6 (13.6) | 1.603 | 0.205 |
|  |  |  | Negative | 365 (61.3) | 203 (34.1) | 27 (4.5) |  |  | 933 (78.4) | 257 (21.6) |  |  |
|  |  | Pericarditis | Positive | 10 (62.5) | 4 (25.0) | 2 (12.5) | --- | 0.214^a^ | 24 (75.0) | 8 (25.0) | 0.266 | 0.606 |
|  |  |  | Negative | 371 (61.7) | 205 (34.1) | 25 (4.2) |  |  | 947 (78.8) | 255 (21.2) |  |  |
|  |  | Renal disorder | Positive | 47 (60.3) | 27 (34.6) | 4 (5.1) | 0.162 | 0.922 | 121 (77.6) | 35 (22.4) | 0.134 | 0.714 |
|  |  |  | Negative | 334 (62.0) | 182 (33.8) | 23 (4.3) |  |  | 850 (78.8) | 228 (21.2) |  |  |
|  |  | Neurological disorder | Positive | 15 (68.2) | 6 (27.3) | 1 (4.5) | 0.448 | 0.799 | 36 (81.8) | 8 (18.2) | 0.267 | 0.606 |
|  |  |  | Negative | 366 (61.5) | 203 (34.1) | 26 (4.4) |  |  | 935 (78.6) | 255 (21.4) |  |  |
|  |  | SLEDAI | More active (SLEDAI＞10) | 27 (71.1) | 9 (23.7) | 2 (5.2) | --- | 0.171^a^ | 63 (82.9) | 13 (17.1) | 0.727 | 0.394 |
|  |  |  | Less active (SLEDAI≤10) | 31 (57.4) | 22 (40.7) | 1 (1.9) |  |  | 84 (77.8) | 24 (22.2) |  |  |

n, number; SNP, single-nucleotide polymorphism; M, major alleles; m, minor alleles; SLEDAI, systemic lupus erythematosus disease activity index.

* The *p* values are not corrected for multiple testings, Bonferroni corrected *p* = 0.0167; ^a^ Calculated by Fisher’ exact test (exact *p* value).
