# Supplementary material for: Association of leptin and leptin receptor gene polymorphisms with systemic lupus erythematosus in a Chinese population
Source: J Cell Mol Med. 2017 Feb 28;21(9):1732–41. doi: 10.1111/jcmm.13093 (PMC5571531; doi:10.1111/jcmm.13093)
Supplement: Supplementary file 5 — Table S5 Association of clinical characteristics with genotype and allele frequencies in LEPR [file JCMM-21-1732-s005.doc]

**Table S5** Association of clinical characteristics with genotype and allele frequencies in *LEPR*

| Gene (SNP) | Allele | Clinical features | Group | Genetypes n (%) | | | χ2 | *P* value* | Alleles n (%) | | χ2 | *P* value |
| --- | --- | --- | --- | --- | --- | --- | --- | --- | --- | --- | --- | --- |
|  | (M/m) |  |  | MM | Mm | mm |  |  | M | m |  |  |
| rs10749754 | A/G | Malar rash | Positive | 166 (73.8) | 55 (24.4) | 4 (1.8) | 0.278 | 0.870 | 387 (86.0) | 63 (14.0) | 0.132 | 0.716 |
|  |  |  | Negative | 293 (74.7) | 94 (24.0) | 5 (1.3) |  |  | 680 (86.7) | 104 (13.3) |  |  |
|  |  | Discoid rash | Positive | 49 (75.4) | 15 (23.1) | 1 (1.5) | 0.047 | 0.977 | 113 (86.9) | 17 (13.1) | 0.026 | 0.872 |
|  |  |  | Negative | 410 (74.3) | 134 (24.3) | 8 (1.4) |  |  | 954 (86.4) | 150 (13.6) |  |  |
|  |  | Photosensitivity | Positive | 39 (68.4) | 17 (29.8) | 1 (1.8) | 1.176 | 0.555 | 95 (83.3) | 19 (16.7) | 1.054 | 0.305 |
|  |  |  | Negative | 420 (75.0) | 132 (23.6) | 8 (1.4) |  |  | 972 (86.8) | 148 (13.2) |  |  |
|  |  | Oral ulcers | Positive | 50 (67.6) | 21 (28.4) | 3 (4.1) | 5.032 | 0.081 | 121 (81.8) | 27 (18.2) | 3.188 | 0.074 |
|  |  |  | Negative | 409 (75.3) | 128 (23.6) | 6 (1.1) |  |  | 946 (87.1) | 140 (12.9) |  |  |
|  |  | Arthritis | Positive | 211 (73.5) | 73 (25.4) | 3 (1.0) | 1.051 | 0.591 | 495 (86.2) | 79 (13.8) | 0.048 | 0.826 |
|  |  |  | Negative | 248 (75.2) | 76 (23.0) | 6 (1.8) |  |  | 572 (86.7) | 88 (13.3) |  |  |
|  |  | Pleurisy | Positive | 15 (68.2) | 6 (27.3) | 1 (4.5) | 1.701 | 0.427 | 36 (81.8) | 8 (18.2) | 0.843 | 0.359 |
|  |  |  | Negative | 444 (74.6) | 143 (24.0) | 8 (1.3) |  |  | 1031 (86.6) | 159 (13.4) |  |  |
|  |  | Pericarditis | Positive | 13 (81.3) | 2 (12.5) | 1 (6.3) | --- | 0.119^a^ | 28 (87.5) | 4 (12.5) | --- | 1.000^a^ |
|  |  |  | Negative | 446 (74.2) | 147 (24.5) | 8 (1.3) |  |  | 1039 (86.4) | 163 (13.6) |  |  |
|  |  | Renal disorder | Positive | 57 (73.1) | 19 (24.4) | 2 (2.6) | 0.770 | 0.680 | 133 (85.3) | 23 (14.7) | 0.224 | 0.636 |
|  |  |  | Negative | 402 (74.6) | 130 (24.1) | 7 (1.3) |  |  | 934 (86.6) | 144 (13.4) |  |  |
|  |  | Neurological disorder | Positive | 18 (81.8) | 4 (18.2) | 0 (0.00) | --- | 0.725^a^ | 40 (90.9) | 4 (9.1) | --- | 0.503^a^ |
|  |  |  | Negative | 441 (74.1) | 145 (24.4) | 9 (1.5) |  |  | 1027 (86.3) | 163 (13.7) |  |  |
|  |  | SLEDAI | More active (SLEDAI＞10) | 29 (76.3) | 8 (21.1) | 1 (2.6) | --- | 0.854^a^ | 66 (86.8) | 10 (13.2) | 0.101 | 0.751 |
|  |  |  | Less active (SLEDAI≤10) | 41 (75.9) | 10 (18.5) | 3 (5.6) |  |  | 92 (85.2) | 16 (14.8) |  |  |
| rs1137100 | G/A | Malar rash | Positive | 153 (68.0) | 56 (24.9) | 16 (7.1) | 5.419 | 0.067 | 362 (80.4) | 88 (19.6) | 2.239 | 0.135 |
|  |  |  | Negative | 277 (70.7) | 103 (26.3) | 12 (3.0) |  |  | 657 (83.8) | 127 (16.2) |  |  |
|  |  | Discoid rash | Positive | 48 (73.8) | 15 (23.1) | 2 (3.1) | 0.726 | 0.696 | 111 (85.4) | 19 (14.6) | 0.796 | 0.372 |
|  |  |  | Negative | 382 (69.2) | 144 (26.1) | 26 (4.7) |  |  | 908 (82.2) | 196 (17.8) |  |  |
|  |  | Photosensitivity | Positive | 34 (59.6) | 17 (29.8) | 6 (10.5) | 6.272 | **0.043** | 85 (74.6) | 29 (25.4) | 5.609 | **0.018** |
|  |  |  | Negative | 396 (70.7) | 142 (25.4) | 22 (3.9) |  |  | 934 (83.4) | 186 (16.6) |  |  |
|  |  | Oral ulcers | Positive | 54 (73.0) | 17 (23.0) | 3 (4.1) | 0.429 | 0.807 | 125 (84.5) | 23 (15.5) | 0.414 | 0.520 |
|  |  |  | Negative | 376 (69.2) | 142 (26.2) | 25 (4.6) |  |  | 894 (82.3) | 192 (17.7) |  |  |
|  |  | Arthritis | Positive | 197 (68.6) | 80 (27.9) | 10 (3.5) | 2.320 | 0.313 | 474 (82.6) | 100 (17.4) | 0.000 | 0.999 |
|  |  |  | Negative | 233 (70.6) | 79 (23.9) | 18 (5.5) |  |  | 545 (82.6) | 115 (17.4) |  |  |
|  |  | Pleurisy | Positive | 16 (72.7) | 5 (22.7) | 1 (4.5) | 0.112 | 0.945 | 37 (84.1) | 7 (15.9) | 0.073 | 0.787 |
|  |  |  | Negative | 414 (69.6) | 154 (25.9) | 27 (4.7) |  |  | 982 (82.5) | 208 (17.5) |  |  |
|  |  | Pericarditis | Positive | 12 (75.0) | 2 (12.5) | 2 (12.5) | --- | 0.131^a^ | 26 (81.3) | 6 (18.7) | 0.040 | 0.841 |
|  |  |  | Negative | 418 (69.6) | 157 (26.1) | 26 (4.3) |  |  | 993 (82.6) | 209 (17.4) |  |  |
|  |  | Renal disorder | Positive | 55 (70.5) | 20 (25.6) | 3 (3.8) | 0.103 | 0.950 | 130 (83.3) | 26 (16.7) | 0.071 | 0.790 |
|  |  |  | Negative | 375 (69.6) | 139 (25.8) | 25 (4.6) |  |  | 889 (82.5) | 189 (17.5) |  |  |
|  |  | Neurological disorder | Positive | 17 (77.3) | 5 (22.7) | 0 (0.00) | --- | 0.596^a^ | 39 (88.6) | 5 (11.4) | 1.164 | 0.281 |
|  |  |  | Negative | 413 (69.4) | 154 (25.9) | 28 (4.7) |  |  | 980 (82.4) | 210 (17.6) |  |  |
|  |  | SLEDAI | More active (SLEDAI＞10) | 28 (73.7) | 8 (21.1) | 2 (5.3) | --- | 0.936^a^ | 64 (84.2) | 12 (15.8) | 0.025 | 0.874 |
|  |  |  | Less active (SLEDAI≤10) | 40 (74.1) | 10 (18.5) | 4 (7.4) |  |  | 90 (83.3) | 18 (16.7) |  |  |
| rs1137101 | G/A | Malar rash | Positive | 168 (74.7) | 51 (22.7) | 6 (2.7) | 2.489 | 0.288 | 387 (86.0) | 63 (14.0) | 0.908 | 0.341 |
|  |  |  | Negative | 301 (76.8) | 87 (22.2) | 4 (1.0) |  |  | 689 (87.9) | 95 (12.1) |  |  |
|  |  | Discoid rash | Positive | 52 (80.0) | 12 (18.5) | 1 (1.5) | 0.650 | 0.722 | 116 (89.2) | 14 (10.8) | 0.539 | 0.463 |
|  |  |  | Negative | 417 (75.5） | 126 (22.8) | 9 (1.6) |  |  | 960 (87.0) | 144 (13.0) |  |  |
|  |  | Photosensitivity | Positive | 41 (71.9) | 15 (26.3) | 1 (1.8) | 0.583 | 0.747 | 97 (85.1) | 17 (14.9) | 0.500 | 0.479 |
|  |  |  | Negative | 428 (76.4) | 123 (22.0) | 9 (1.6) |  |  | 979 (87.4) | 141 (12.6) |  |  |
|  |  | Oral ulcers | Positive | 53 (71.6) | 18 (24.3) | 3 (4.1) | 3.429 | 0.180 | 124 (83.8) | 24 (16.2) | 1.754 | 0.185 |
|  |  |  | Negative | 416 (76.6) | 120 (22.1) | 7 (1.3) |  |  | 952 (87.7) | 134 (12.3) |  |  |
|  |  | Arthritis | Positive | 218 (76.0) | 64 (22.3) | 5 (1.7) | 0.050 | 0.975 | 500 (87.1) | 74 (12.9) | 0.007 | 0.931 |
|  |  |  | Negative | 251 (76.1) | 74 (22.4) | 5 (1.5) |  |  | 576 (87.3) | 84 (12.7) |  |  |
|  |  | Pleurisy | Positive | 16 (72.7) | 5 (22.7) | 1 (4.5) | 1.238 | 0.539 | 37 (84.1) | 7 (15.9) | 0.394 | 0.530 |
|  |  |  | Negative | 453 (76.1) | 133 (22.4) | 9 (1.5) |  |  | 1039 (87.3) | 151 (12.7) |  |  |
|  |  | Pericarditis | Positive | 13 (81.3) | 2 (12.5) | 1 (6.3) | --- | 0.186^a^ | 28 (87.5) | 4 (12.5) | --- | 1.000^a^ |
|  |  |  | Negative | 456 (75.9) | 136 (22.6) | 9 (1.5) |  |  | 1048 (87.2) | 154 (12.8) |  |  |
|  |  | Renal disorder | Positive | 60 (76.9) | 17 (21.8) | 1 (0.00) | 0.086 | 0.958 | 137 (87.8) | 19 (12.2) | 0.062 | 0.803 |
|  |  |  | Negative | 409 (75.9) | 121 (22.4) | 9 (1.7) |  |  | 939 (87.1) | 139 (12.9) |  |  |
|  |  | Neurological disorder | Positive | 20 (90.9) | 2 (9.1) | 0 (0) | --- | 0.186^a^ | 42 (95.5) | 2 (4.5) | --- | 0.109^a^ |
|  |  |  | Negative | 449 (75.5) | 136 (22.9) | 10 (1.7) |  |  | 1034 (86.9) | 156 (13.1) |  |  |
|  |  | SLEDAI | More active (SLEDAI＞10) | 31 (81.6) | 6 (15.8) | 1 (2.6) | --- | 0.907^a^ | 68 (89.5) | 8 (10.5) | 0.252 | 0.616 |
|  |  |  | Less active (SLEDAI≤10) | 42 (77.8) | 10 (18.5) | 2 (3.7) |  |  | 94 (87.0) | 14 (13.0) |  |  |
| rs13306519 | C/G | Malar rash | Positive | 156 (69.3) | 57 (25.3) | 12 (5.3) | 0.923 | 0.630 | 369 (82.0) | 81 (18.0) | 0.849 | 0.357 |
|  |  |  | Negative | 257 (65.6) | 112 (28.6) | 23 (5.9) |  |  | 626 (79.8) | 158 (20.2) |  |  |
|  |  | Discoid rash | Positive | 40 (61.5) | 22 (33.8) | 3 (4.6) | 1.565 | 0.457 | 102 (78.5) | 28 (21.5) | 0.438 | 0.508 |
|  |  |  | Negative | 373 (67.6) | 147 (26.6) | 32 (5.8) |  |  | 893 (80.9) | 211 (19.1) |  |  |
|  |  | Photosensitivity | Positive | 39 (68.4) | 15 (26.3) | 3 (5.3) | 0.066 | 0.968 | 93 (81.6) | 21 (18.4) | 0.072 | 0.788 |
|  |  |  | Negative | 374 (66.8) | 154 (27.5) | 32 (5.7) |  |  | 902 (80.5) | 218 (19.5) |  |  |
|  |  | Oral ulcers | Positive | 46 (62.2) | 25 (33.8) | 3 (4.1) | 1.929 | 0.381 | 117 (79.1) | 31 (20.9) | 0.268 | 0.605 |
|  |  |  | Negative | 367 (67.6) | 144 (26.5) | 32 (5.9) |  |  | 878 (80.8) | 208 (19.2) |  |  |
|  |  | Arthritis | Positive | 186 (64.8) | 85 (29.6) | 16 (5.6) | 1.343 | 0.511 | 457 (79.6) | 117 (20.4) | 0.708 | 0.400 |
|  |  |  | Negative | 227 (68.7) | 84 (25.5) | 19 (5.8) |  |  | 538 (81.5) | 122 (18.5) |  |  |
|  |  | Pleurisy | Positive | 17 (77.3) | 5 (22.7) | 0 (0.00) | --- | 0.426^a^ | 39 (88.6) | 5 (11.4) | 1.872 | 0.171 |
|  |  |  | Negative | 396 (66.6) | 164 (27.6) | 35 (5.9) |  |  | 956 (80.3) | 234 (19.7) |  |  |
|  |  | Pericarditis | Positive | 13 (81.3) | 3 (18.8) | 0 (0.00) | --- | 0.341^a^ | 29 (90.6) | 3 (9.4) | --- | 0.178^a^ |
|  |  |  | Negative | 400 (66.6) | 166 (27.6) | 35 (5.8) |  |  | 966 (80.4) | 236 (19.6) |  |  |
|  |  | Renal disorder | Positive | 49 (62.8) | 20 (25.6) | 9 (11.5) | 5.742 | 0.057 | 118 (75.6) | 38 (24.4) | 2.849 | 0.091 |
|  |  |  | Negative | 364 (67.5) | 149 (27.6) | 26 (4.8) |  |  | 877 (81.4) | 201 (18.6) |  |  |
|  |  | Neurological disorder | Positive | 15 (68.2) | 5 (22.7) | 2 (9.1) | 0.656 | 0.720 | 35 (79.5) | 9 (20.5) | 0.034 | 0.853 |
|  |  |  | Negative | 398 (66.9) | 164 (27.6) | 33 (5.5) |  |  | 960 (80.7) | 230 (19.3) |  |  |
|  |  | SLEDAI | More active (SLEDAI＞10) | 26 (68.4) | 8 (21.1) | 4 (10.5) | --- | 0.649^a^ | 60 (78.9) | 16 (21.1) | 0.347 | 0.556 |
|  |  |  | Less active (SLEDAI≤10) | 38 (70.4) | 13 (24.1) | 3 (5.6) |  |  | 89 (82.4) | 19 (17.6) |  |  |
| rs8179183 | C/G | Malar rash | Positive | 208 (92.4) | 17 (7.6) | 0 (0.00) | --- | 0.779^a^ | 433 (96.2) | 17 (3.8) | 0.461 | 0.497 |
|  |  |  | Negative | 357 (91.1) | 34 (8.7) | 1 (0.3) |  |  | 748 (95.4) | 36 (4.6) |  |  |
|  |  | Discoid rash | Positive | 62 (95.4) | 3 (4.6) | 0 (0.00) | --- | 0.413^a^ | 127 (97.7) | 3 (2.3) | --- | 0.237^a^ |
|  |  |  | Negative | 503 (91.1) | 48 (8.7) | 1 (0.2) |  |  | 1054 (95.5) | 50 (4.5) |  |  |
|  |  | Photosensitivity | Positive | 53 (93.0) | 4 (7.0) | 0 (0.00) | --- | 0.826^a^ | 110 (96.5) | 4 (3.5) | --- | 0.812^a^ |
|  |  |  | Negative | 512 (91.4) | 47 (8.4) | 1 (0.2) |  |  | 1071 (95.6) | 49 (4.4) |  |  |
|  |  | Oral ulcers | Positive | 66 (89.2) | 8 (10.8) | 0 (0.00) | --- | 0.559^a^ | 140 (94.6) | 8 (5.4) | 0.504 | 0.478 |
|  |  |  | Negative | 499 (91.9) | 43 (7.9) | 1 (0.2) |  |  | 1041 (95.9) | 45 (4.1) |  |  |
|  |  | Arthritis | Positive | 263 (91.6) | 23 (8.0) | 1 (0.3) | --- | 0.710^a^ | 549 (95.6) | 25 (4.4) | 0.010 | 0.922 |
|  |  |  | Negative | 302 (91.5) | 28 (8.5) | 0 (0.00) |  |  | 632 (95.8) | 28 (4.2) |  |  |
|  |  | Pleurisy | Positive | 21 (95.5) | 1 (4.5) | 0 (0.00) | --- | 0.724^a^ | 43 (97.7) | 1 (2.3) | --- | 0.500^a^ |
|  |  |  | Negative | 544 (91.4) | 50 (8.4) | 1 (0.2) |  |  | 1138 (95.6) | 52 (4.4) |  |  |
|  |  | Pericarditis | Positive | 14 (87.5) | 2 (12.5) | 0 (0.00) | --- | 0.644^a^ | 30 (93.7) | 2 (6.3) | --- | 0.645^a^ |
|  |  |  | Negative | 551 (91.7) | 49 (8.2) | 1 (0.2) |  |  | 1151 (95.8) | 51 (4.2) |  |  |
|  |  | Renal disorder | Positive | 73 (93.6) | 5 (6.4) | 0 (0.00) | --- | 0.705^a^ | 151 (96.8) | 5 (3.2) | 0.516 | 0.473 |
|  |  |  | Negative | 492 (91.3) | 46 (8.5) | 1 (0.2) |  |  | 1030 (95.5) | 48 (4.5) |  |  |
|  |  | Neurological disorder | Positive | 21 (95.5) | 1 (4.5) | 0 (0.00) | --- | 0.724^a^ | 43 (97.7) | 1 (2.3) | --- | 0.500^a^ |
|  |  |  | Negative | 544 (91.4) | 50 (8.4) | 1 (0.2) |  |  | 1138 (95.6) | 52 (4.4) |  |  |
|  |  | SLEDAI | More active (SLEDAI＞10) | 35 (92.1) | 3 (7.9) | 0 (0) | --- | 0.515^a^ | 73 (96.1) | 3 (3.9) | 0.557 | 0.455 |
|  |  |  | Less active (SLEDAI≤10) | 47 (87.0) | 7 (13.0) | 0 (0) |  |  | 101 (93.5) | 7 (6.5) |  |  |
| rs1805096 | A/G | Malar rash | Positive | 166 (73.8) | 55 (24.4) | 4 (1.8) | --- | 0.536^a^ | 387 (86.0) | 63 (14.0) | 1.040 | 0.308 |
|  |  |  | Negative | 302 (77.0) | 86 (21.9) | 4 (1.0) |  |  | 690 (88.0) | 94 (12.0) |  |  |
|  |  | Discoid rash | Positive | 51 (78.5) | 13 (20.0) | 1 (1.5) | 0.357 | 0.837 | 115 (88.5) | 15 (11.5) | 0.184 | 0.668 |
|  |  |  | Negative | 417 (75.5) | 128 (23.2) | 7 (1.3) |  |  | 962 (87.1) | 142 (12.9) |  |  |
|  |  | Photosensitivity | Positive | 40 (70.2) | 15 (26.3) | 2 (3.5) | 2.867 | 0.227 | 95 (83.3) | 19 (16.7) | 1.759 | 0.185 |
|  |  |  | Negative | 428 (76.4) | 126 (22.5) | 6 (1.1) |  |  | 982 (87.7) | 138 (12.3) |  |  |
|  |  | Oral ulcers | Positive | 55 (74.3) | 18 (24.3) | 1 (1.4) | 0.107 | 0.948 | 128 (86.5) | 20 (13.5) | 0.095 | 0.758 |
|  |  |  | Negative | 413 (76.1) | 123 (22.7) | 7 (1.3) |  |  | 949 (87.4) | 137 (12.6) |  |  |
|  |  | Arthritis | Positive | 220 (76.7) | 63 (22.0) | 4 (1.4) | --- | 0.882^a^ | 503 (87.6) | 71 (12.4) | 0.121 | 0.728 |
|  |  |  | Negative | 248 (75.2) | 78 (23.6) | 4 (1.2) |  |  | 574 (87.0) | 86 (13.0) |  |  |
|  |  | Pleurisy | Positive | 16 (72.7) | 5 (22.7) | 1 (4.5) | 1.887 | 0.389 | 37 (84.1) | 7 (15.9) | 0.417 | 0.518 |
|  |  |  | Negative | 452 (76.0) | 136 (22.9) | 7 (1.2) |  |  | 1040 (87.4) | 150 (12.6) |  |  |
|  |  | Pericarditis | Positive | 14 (87.5) | 1 (6.3) | 1 (6.3) | --- | 0.066^a^ | 29 (90.6) | 3 (9.4) | --- | 0.616^a^ |
|  |  |  | Negative | 454 (75.5) | 140 (23.3) | 7 (1.2) |  |  | 1048 (87.2) | 154 (12.8) |  |  |
|  |  | Renal disorder | Positive | 62 (79.5) | 15 (19.2) | 1 (1.3) | 0.668 | 0.716 | 139 (89.1) | 17 (10.9) | 0.536 | 0.464 |
|  |  |  | Negative | 406 (74.7) | 126 (23.8) | 7 (1.4) |  |  | 938 (87.0) | 140 (13.0) |  |  |
|  |  | Neurological disorder | Positive | 18 (81.8) | 3 (13.6) | 1 (4.5) | --- | 0.223^a^ | 39 (88.6) | 5 (11.4) | 0.076 | 0.783 |
|  |  |  | Negative | 450 (75.6) | 138 (23.1) | 7 (1.2) |  |  | 1038 (87.2) | 152 (12.8) |  |  |
|  |  | SLEDAI | More active (SLEDAI＞10) | 31 (81.6) | 6 (15.8) | 1 (2.6) | --- | 0.568 | 68 (89.5) | 8 (10.5) | 0.006 | 0.940 |
|  |  |  | Less active (SLEDAI≤10) | 43 (79.6) | 11 (20.4) | 0 (0) |  |  | 97 (89.8) | 11 (10.2) |  |  |
| rs3790434 | C/T | Malar rash | Positive | 148 (65.8) | 73 (32.4) | 4 (1.8) | 3.063 | 0.216 | 369 (82.0) | 81 (18.0) | 2.776 | 0.096 |
|  |  |  | Negative | 284 (72.4) | 103 (26.3) | 5 (1.3) |  |  | 671 (85.6) | 113 (14.4) |  |  |
|  |  | Discoid rash | Positive | 38 (58.5) | 26 (40.0) | 1 (1.5) | 4.742 | 0.093 | 102 (78.5) | 28 (21.5) | 3.711 | 0.054 |
|  |  |  | Negative | 394 (71.4) | 150 (27.2) | 8 (1.4) |  |  | 938 (85.0) | 166 (15.0) |  |  |
|  |  | Photosensitivity | Positive | 34 (59.6) | 22 (38.6) | 1 (1.8) | 3.235 | 0.198 | 90 (78.9) | 24 (21.1) | 2.695 | 0.101 |
|  |  |  | Negative | 398 (71.1) | 154 (27.5) | 8 (1.4) |  |  | 950 (84.8) | 170 (15.2) |  |  |
|  |  | Oral ulcers | Positive | 51 (68.9) | 23 (31.1) | 0 (0.00) | --- | 0.485^a^ | 125 (84.5) | 23 (15.5) | 0.004 | 0.949 |
|  |  |  | Negative | 381 (70.2) | 153 (28.2) | 9 (1.7) |  |  | 915 (84.3) | 171 (15.7) |  |  |
|  |  | Arthritis | Positive | 200 (69.7) | 80 (27.9) | 7 (2.4) | 3.624 | 0.163 | 480 (83.6) | 94 (16.4) | 0.348 | 0.555 |
|  |  |  | Negative | 232 (70.3) | 96 (29.1) | 2 (0.6) |  |  | 560 (84.8) | 100 (15.2) |  |  |
|  |  | Pleurisy | Positive | 16 (72.7) | 6 (27.3) | 0 (0.00) | --- | 0.831^a^ | 38 (86.4) | 6 (13.6) | 0.150 | 0.699 |
|  |  |  | Negative | 416 (69.9) | 170 (28.6) | 9 (1.5) |  |  | 1002 (84.2) | 188 (15.8) |  |  |
|  |  | Pericarditis | Positive | 12 (75.0) | 4 (25.0) | 0 (0.00) | --- | 0.831^a^ | 28 (87.5) | 4 (12.5) | --- | 0.644^a^ |
|  |  |  | Negative | 420 (69.9) | 172 (28.6) | 9 (1.5) |  |  | 1012 (84.2) | 190 (15.8) |  |  |
|  |  | Renal disorder | Positive | 54 (69.2) | 23 (29.5) | 1 (1.3) | 0.056 | 0.972 | 131 (84.0) | 25 (16.0) | 0.012 | 0.911 |
|  |  |  | Negative | 378 (28.4) | 153 (28.4) | 8 (1.5) |  |  | 909 (84.3) | 169 (15.7) |  |  |
|  |  | Neurological disorder | Positive | 13 (59.1) | 8 (36.4) | 1 (4.5) | 2.371 | 0.306 | 34 (77.3) | 10 (22.7) | 1.690 | 0.194 |
|  |  |  | Negative | 419 (70.4) | 168 (28.2) | 8 (1.3) |  |  | 1006 (84.5) | 184 (15.5) |  |  |
|  |  | SLEDAI | More active (SLEDAI＞10) | 23 (60.5) | 14 (36.8) | 1 (2.6) | --- | 0.643^a^ | 60 (78.9) | 16 (21.1) | 1.208 | 0.272 |
|  |  |  | Less active (SLEDAI≤10) | 39 (72.2) | 14 (25.9) | 1 (1.9) |  |  | 92 (85.2) | 16 (14.8) |  |  |
| rs3806318 | A/G | Malar rash | Positive | 181 (80.4) | 40 (17.8) | 4 (1.8) | 0.251 | 0.882 | 402 (89.3) | 48 (10.7) | 0.066 | 0.798 |
|  |  |  | Negative | 317 (80.9) | 70 (17.9) | 5 (1.3) |  |  | 704 (89.8) | 80 (10.2) |  |  |
|  |  | Discoid rash | Positive | 53 (81.5) | 12 (18.5) | 0 (0.00) | --- | 0.726^a^ | 118 (90.8) | 12 (9.2) | 0.204 | 0.652 |
|  |  |  | Negative | 445 (80.6) | 98 (17.8) | 9 (1.6) |  |  | 988 (89.5) | 116 (10.5) |  |  |
|  |  | Photosensitivity | Positive | 40 (70.2) | 14 (24.6) | 3 (5.3) | 8.693 | **0.013** | 94 (82.5) | 20 (17.5) | 6.948 | **0.008** |
|  |  |  | Negative | 458 (81.8) | 96 (17.1) | 6 (1.1) |  |  | 1012 (90.4) | 108 (9.6) |  |  |
|  |  | Oral ulcers | Positive | 59 (79.7) | 15 (20.3) | 0 (0.00) | --- | 0.468^a^ | 133 (89.9) | 15 (10.1) | 0.010 | 0.919 |
|  |  |  | Negative | 439 (80.8) | 95 (17.5) | 9 (1.7) |  |  | 973 (89.6) | 113 (10.4) |  |  |
|  |  | Arthritis | Positive | 237 (82.6) | 43 (15.0) | 7 (2.4) | 6.204 | **0.045** | 517 (90.1) | 57 (9.9) | 0.226 | 0.635 |
|  |  |  | Negative | 261 (79.1) | 67 (20.3) | 2 (0.6) |  |  | 589 (89.2) | 71 (10.8) |  |  |
|  |  | Pleurisy | Positive | 16 (72.7) | 5 (22.7) | 1 (4.5) | 1.978 | 0.372 | 37 (84.1) | 7 (15.9) | 1.504 | 0.220 |
|  |  |  | Negative | 482 (81.0) | 105 (17.6) | 8 (1.3) |  |  | 1069 (89.8) | 121 (10.2) |  |  |
|  |  | Pericarditis | Positive | 14 (87.5) | 2 (12.5) | 0 (0.00) | --- | 0.802^a^ | 30 (93.7) | 2 (6.3) | --- | 0.570^a^ |
|  |  |  | Negative | 484 (80.5) | 108 (18.0) | 9 (1.5) |  |  | 1076 (89.5) | 126 (10.5) |  |  |
|  |  | Renal disorder | Positive | 65 (83.3) | 11 (14.1) | 2 (2.6) | 1.519 | 0.468 | 141 (90.4) | 15 (9.6) | 0.110 | 0.740 |
|  |  |  | Negative | 433 (80.3) | 99 (18.4) | 7 (1.3) |  |  | 965 (89.5) | 113 (10.5) |  |  |
|  |  | Neurological disorder | Positive | 20 (90.9) | 2 (9.1) | 0 (0.00) | --- | 0.514^a^ | 42 (95.5) | 2 (4.5) | --- | 0.224^a^ |
|  |  |  | Negative | 478 (80.3) | 108 (18.2) | 9 (1.5) |  |  | 1064 (89.4) | 126 (10.6) |  |  |
|  |  | SLEDAI | More active (SLEDAI＞10) | 33 (86.8) | 3 (7.9) | 2 (5.3) | --- | **0.020 ^a^** | 69 (90.8) | 7 (9.2) | 2.112 | 0.146 |
|  |  |  | Less active (SLEDAI≤10) | 37 (68.5) | 16 (29.6) | 1 (1.9) |  |  | 90 (83.3) | 18 (16.7) |  |  |
| rs7518632 | A/C | Malar rash | Positive | 143 (63.6) | 66 (29.3) | 16 (7.1) | 3.571 | 0.168 | 352 (78.2) | 98 (21.8) | 0.459 | 0.498 |
|  |  |  | Negative | 249 (63.5) | 128 (32.7) | 15 (3.8) |  |  | 626 (79.8) | 158 (20.2) |  |  |
|  |  | Discoid rash | Positive | 46 (70.8) | 16 (24.6) | 3 (4.6) | 1.700 | 0.427 | 108 (83.1) | 22 (16.9) | 1.291 | 0.256 |
|  |  |  | Negative | 346 (62.7) | 178 (32.2) | 28 (5.1) |  |  | 870 (78.8) | 234 (21.2) |  |  |
|  |  | Photosensitivity | Positive | 34 (59.6) | 18 (31.6) | 5 (8.8) | 1.905 | 0.386 | 86 (75.4) | 28 (24.6) | 1.112 | 0.292 |
|  |  |  | Negative | 358 (63.9) | 176 (31.4) | 26 (4.6) |  |  | 892 (79.6) | 228 (20.4) |  |  |
|  |  | Oral ulcers | Positive | 47 (63.5) | 22 (29.7) | 5 (6.8) | 0.581 | 0.748 | 116 (78.4) | 32 (21.6) | 0.079 | 0.779 |
|  |  |  | Negative | 345 (63.5) | 172 (31.7) | 26 (4.8) |  |  | 862 (79.4) | 224 (20.6) |  |  |
|  |  | Arthritis | Positive | 184 (64.1) | 94 (32.8) | 9 (3.1) | 4.130 | 0.127 | 462 (80.5) | 112 (19.5) | 0.993 | 0.319 |
|  |  |  | Negative | 208 (63.0) | 100 (30.3) | 22 (6.7) |  |  | 516 (78.2) | 144 (21.8) |  |  |
|  |  | Pleurisy | Positive | 15 (68.2) | 6 (27.3) | 1 (4.5) | 0.214 | 0.898 | 36 (81.8) | 4 (18.2) | --- | 0.111^a^ |
|  |  |  | Negative | 377 (63.4) | 188 (31.6) | 30 (5.0) |  |  | 942 (79.2) | 248 (20.8) |  |  |
|  |  | Pericarditis | Positive | 12 (75.0) | 3 (18.8) | 1 (6.3) | --- | 0.601^a^ | 27 (84.4) | 5 (15.6) | 0.542 | 0.469 |
|  |  |  | Negative | 380 (63.2) | 191 (31.8) | 30 (5.0) |  |  | 951 (79.1) | 251 (20.9) |  |  |
|  |  | Renal disorder | Positive | 55 (70.5) | 20 (25.6) | 3 (3.8) | 1.887 | 0.389 | 130 (83.3) | 26 (16.7) | 1.807 | 0.179 |
|  |  |  | Negative | 337 (62.5) | 174 (32.3) | 28 (5.2) |  |  | 848 (78.7) | 230 (21.3) |  |  |
|  |  | Neurological disorder | Positive | 17 (77.3) | 4 (18.2) | 1 (4.5) | --- | 0.367^a^ | 38 (86.4) | 6 (13.6) | 1.403 | 0.236 |
|  |  |  | Negative | 375 (63.0) | 190 (31.9) | 30 (5.0) |  |  | 940 (79.0) | 250 (21.0) |  |  |
|  |  | SLEDAI | More active (SLEDAI＞10) | 27 (71.1) | 9 (23.7) | 2 (5.3) | 2.306 | 0.351 | 63 (82.9) | 13 (17.1) | 0.309 | 0.578 |
|  |  |  | Less active (SLEDAI≤10) | 39 (72.2) | 8 (14.2) | 7 (13.0) |  |  | 86 (79.6) | 22 (20.4) |  |  |

n, number; SNP, single-nucleotide polymorphism; M, major alleles; m, minor alleles; SLEDAI, systemic lupus erythematosus disease activity index.

* The *p* values are not corrected for multiple testings, Bonferroni corrected *p* = 0.0167; ^a^ Calculated by Fisher’ exact test (exact *p* value).
